# Comparison of intensity normalization methods in prostate, brain, and breast cancer multi-parametric magnetic resonance imaging

**DOI:** 10.3389/fonc.2025.1433444

**Published:** 2025-02-07

**Authors:** Savannah R. Duenweg, Samuel A. Bobholz, Allison K. Lowman, Aleksandra Winiarz, Biprojit Nath, Michael J. Barrett, Fitzgerald Kyereme, Stephanie Vincent-Sheldon, Peter LaViolette

**Affiliations:** ^1^ Department of Biophysics, Medical College of Wisconsin, Milwaukee, WI, United States; ^2^ Department of Radiology, Medical College of Wisconsin, Milwaukee, WI, United States

**Keywords:** MRI, prostate cancer, brain cancer, breast cancer, normalization, radiomics

## Abstract

**Objectives:**

Intensity variation in multi-parametric magnetic resonance imaging (MP-MRI) is a confounding factor in MRI analyses. Previous studies have employed several normalization methods, but there is a lack of consensus on which method results in the most comparable images across vendors and acquisitions. This study used MP-MRI collected from patients with confirmed prostate, brain, or breast cancer to examine common intensity normalization methods to identify which best harmonizes intensity values across cofounds.

**Materials and methods:**

Multiple normalization methods were deployed for intensity comparison between three unique sites, MR vendors, and magnetic field strength. Additionally, we calculated radiomic features before and after intensity normalization to determine how downstream analyses may be affected. Specifically, in the prostate cancer cohort, we tested these methods on T2-weighted imaging (T2WI) and additionally looked at a subset of patients who were scanned with and without the use of an endorectal coil (ERC). In a cohort of glioblastoma (GBM) patients, we tested these methods in T1 pre- and post-contrast enhancement (T1, T1C), fluid attenuated inversion recovery (FLAIR), and apparent diffusion coefficient (ADC) maps. Finally, in the breast cancer cohort, we tested methods on T1-weighted nonfat-suppressed images. All methods were compared using a two one-sided test (TOST) to test for equivalence of mean and standard deviation of intensity distributions.

**Results:**

While each organ had unique results, across every tested comparison, using the Z-score of intensity within a mask of the organ consistently provided an equivalent distribution (all p < 0.001).

**Conclusions:**

Our results suggest that intensity normalization using the Z-score of intensity within prostate, breast, and brain MR images produces the most comparable intensities between sites, MR vendors, magnetic field strength, and prostate endorectal coil usage. Likewise, Z-score normalization provided the highest percentage of radiomic features that were statistically equal across the three organs.

## 1 Introduction

Multi-parametric magnetic resonance imaging (MP-MRI) is used to assess cancer and response to therapy. Specific to prostate cancer, a typical MP-MRI protocol contains T2-weighted (T2W), diffusion-weighted (DWI), and dynamic contrast enhanced (DCE) imaging. The Prostate and Breast Imaging Reporting and Data Systems, PI-RADS and BI-RADS, respectively, assign a score to MR images and have standardized acquisition, interpretation, and reporting of prostate and breast MRI, as well as aid in the accurate detection of cancerous lesions (1). Moreover, MP-MRI including T1-weighted imaging pre- and post-gadolinium contrast agent (T1 and T1C, respectively) is used to maximize the efficiency of surgical resection and radiation treatment, as well as monitoring progression, for glioblastoma.

While MP-MRI acquisitions are well established techniques for imaging several organs, voxel intensities in “weighted” scans are nonquantitative and can vary within and across patients, tissues, and MRI vendors. Clinically, the most used MRI acquisitions include pre- and post-contrast T1-weighted, T2-weighted, and diffusion weighted imaging (DWI). These scans are assessed qualitatively to determine cancer presence, although apparent diffusion coefficient maps (ADC) can be created from DWI for quantitative assessment. Acquisitions including MR fingerprinting (MRF), advanced diffusion, and a variety of quantitative MRI (QMRI) have been an area of interest for both response assessments in clinical trials and multi-institutional studies. These acquisitions however are not used clinically due to long scan times and variability in acquisition parameters and post-processing techniques (2–4).

To make inter- and intra-patient quantitative comparisons, such as with radiomic analyses, images need to be intensity normalized as a pre-processing step. Furthermore, normalization is necessary for the development of MRI-based machine learning techniques for diagnosis of cancer. There is no current gold standard method for signal intensity normalization, however, a previously published paper by Shinohara et al. (5) discussed seven statistical principles of imaging normalization including: (1) common interpretation across locations within the same tissue type, (2) replicability, (3) preservation of rank intensities, (4) similar distributions within and across patients, (5) uninfluenced by biological abnormality or population heterogeneity, (6) minimal sensitivity to noise and artifacts, and (7) do not result in a loss of information associated with pathology. Prior studies have normalized by average voxel values within fat and muscle tissue regions (6–8), used N4 bias field correction and intensity Z-score (9–11), and histogram matching and mapping techniques to normalize images. Tissue-based normalization has shown to improve inter-patient intensity differences better than unnormalized data and histogram-based normalization methods (12). While Z-score mapping is common among MRI analyses for several disease states (13–16), it can be confounded by factors such as tumor volume and aggressiveness (i.e., increased hypointensity). Additionally, histogram matching and mapping techniques have been shown to be beneficial in normalizing brain MRI (17); however, histogram matching was performed after fat, bone, and background removal, indicating that global normalization of other abdominal organs may be less successful.

Diffusion weighted imaging measures the diffusion of water molecules to generate contrast in MR images. DWI has been shown to detect cancerous tumors and evaluate tumor aggressiveness (4, 18, 19), but much like T1 and T2WI, DWI is also assessed qualitatively by radiologists. Calculation of ADC from multiple b-values allows a quantitative assessment of water diffusion. Previous studies have shown that ADC has an inverse relationship with higher risk prostate, brain, and breast cancers (20–23). While ADC is considered quantitative, factors such as perfusion can affect lower b-values. Previous studies have assessed normalizing ADC maps prior to analysis. One such study found that a signal-to-noise (SNR)-weighted regularization of ADC produced homogenous maps at varying levels of SNR compared to non-regularized maps which could only estimate ADC accurately at high SNR levels (24). Conversely, a study comparing normalizing ADC by the ratio of non-enhancing tumor to normal white matter in high-grade glioma patients showed that normalization did not improve ADC correlations with overall survival (25).

Though the need for intensity normalization is well understood, the lack of normalization standards makes it difficult to compare MRI-based analyses. This study analyzed a variety of imaging acquisitions across multiple organs to determine if a universal normalization method could be applied. Specifically, we assessed T2WI collected from prostate cancer patients; T1, T1C, fluid-attenuated inversion recovery (FLAIR), and ADC images collected from GBM patients; and T1-weighted nonfat-suppressed images (T1nFS) from breast cancer patients across three unique sites, multiple clinical MR vendors, and 1.5T and 3T magnetic field strength to examine commonly used post-acquisition intensity normalization methods to identify which method produces images most comparable across vendors for each tissue. Additionally, we examined T2WI collected from prostate cancer patients with an endorectal coil in place and following ERC removal to determine which normalization method best compares these images. Furthermore, we calculated 218 radiomic features across all images to determine how radiomic features are affected by each normalization method. Overall, we tested the hypothesis that normalizing images using signal intensities within a defined region would produce intensity distributions that are most comparable across sites, MRI vendors, and magnetic field strength than unnormalized data.

## 2 Materials and methods

Data from three unique sites per organ (prostate, glioblastoma, and breast) were assessed for this study. Details from each site are further detailed in the subsequent sections; however, a simplified table of these data sites and organs is provided in **Table 1**.

**Table 1:** Breakdown of prostate, glioblastoma, and breast cancer data by data site, MR manufacturer, and magnetic field strength.

		Demographics		MR Vendor			Magnetic Field Strength	
		Patients	Sex	GE	Siemens	Philips	1.5 T	3T
Prostate	Total	641	M: 641	256	295	90	89	552
	Site 1	385	M: 385	256	125	4	3	382
	Site 2	86	M: 86	0	0	0	86	0
	Site 3	170	M: 170	0	170	86	0	170
Glioblastoma	Total	956	M: 615	408	549	2	53	903
			F: 401					
	Site 1	52	M:35	34	16	2	39	13
			F: 17					
	Site 2	530	M: 320	0	530	–	14	516
			F: 210					
	Site 3	374	M: 222	374	0	–	0	374
			F: 152					
Breast	Total	236	F: 236	190	46	0	185	51
	Site 1	68	F: 68	68	0	–	68	0
	Site 2	100	F: 100	54	46	–	49	51
	Site 3	68	F: 68	68	0	–	68	0

### 2.1 Prostate cancer cohort

#### 2.1.1 Site 1 – local

**Figure 1:**
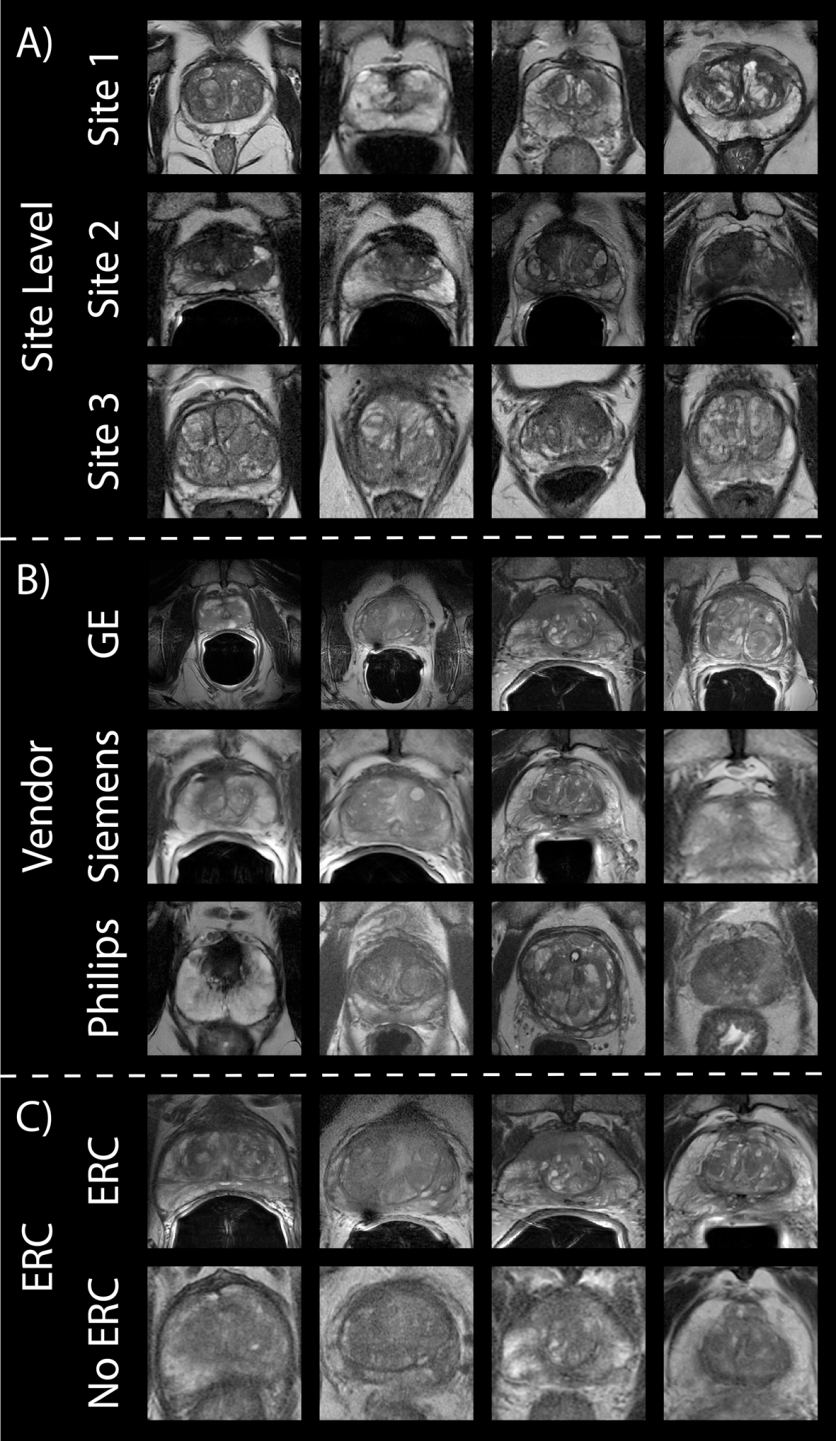
Prostate cancer imaging. Prostate T2WI across **(A)** three data sites, **(B)** three MR vendors (i.e., GE, Siemens, and Philips) and **(C)** with and without an endorectal coil in the subset of Site 1’s patients.

Data from 385 prospectively recruited patients treated locally at our institution (**Table 1** **;** **Figure 1A**, top) with pathologically confirmed prostate cancer undergoing radical prostatectomy between 2014 and 2023 were analyzed for this institutional review board (IRB) approved study. Written informed consent was obtained from all patients. Inclusion criteria for this cohort included clinical imaging including T2-weighted imaging prior to surgery.

Patients underwent multi-parametric magnetic resonance imaging (MP-MRI) prior to prostatectomy on 1.5 T (n_1.5T_ = 3) or 3T (n_3T_ = 382) GE (n_GE_ = 256), Siemens (n_S_ = 125) or Philips (n_P_ = 4) MRI scanner (General Electric, Waukesha, WI, USA; Siemens Healthineers, Erlangen, Germany; Philips, Amsterdam, Netherlands) (**Figure 1B**). A subset of patients (n = 88) had additional imaging after removal of the endorectal coil on either the GE or Siemens scanner (n_GE_ = 69, n_S_ = 19) (**Figure 1C**). Each protocol included T2-weighted imaging with acquisition parameters as follows: repetition time (TR) = 3370 milliseconds, FOV = 120 mm, voxel dimensions = 0.23 × 0.23 × 3 mm, acquisition matrix = 512, and slices = 26. All image contrasts used in this study were acquired axially.

### 2.1.2 Site 2 – PROSTATE-DIAGNOSIS

A publicly available dataset including prostate T2WI scanned on a 1.5 T Philips Achieva using a combined surface and endorectal coil was used for our second site (26, 27). From a total of 92 patients, images from 86 patients were ultimately used in this analysis due to image quality (**Table 1** **;** **Figure 1A**, middle).

#### 2.1.3 Site 3 – PROSTATEx

The final dataset used in this analysis was a collection of retrospective prostate MR studies including T2WI acquired on two different 3T Siemens MR scanners (MAGNETOM Trio and Skyra) (27, 28). T2W imaging acquisition parameters include a turbo spin echo sequence with a resolution of ~0.5 mm in plane and a slice thickness of 3.6 mm. All images were acquired without an endorectal coil. After exclusion of images with poor quality, a total of 170 patients’ images were used (**Table 1** **;** **Figure 1A**, bottom).

### 2.2 Glioblastoma cohort

#### 2.2.1 Site 1 – local

**Figure 2:**
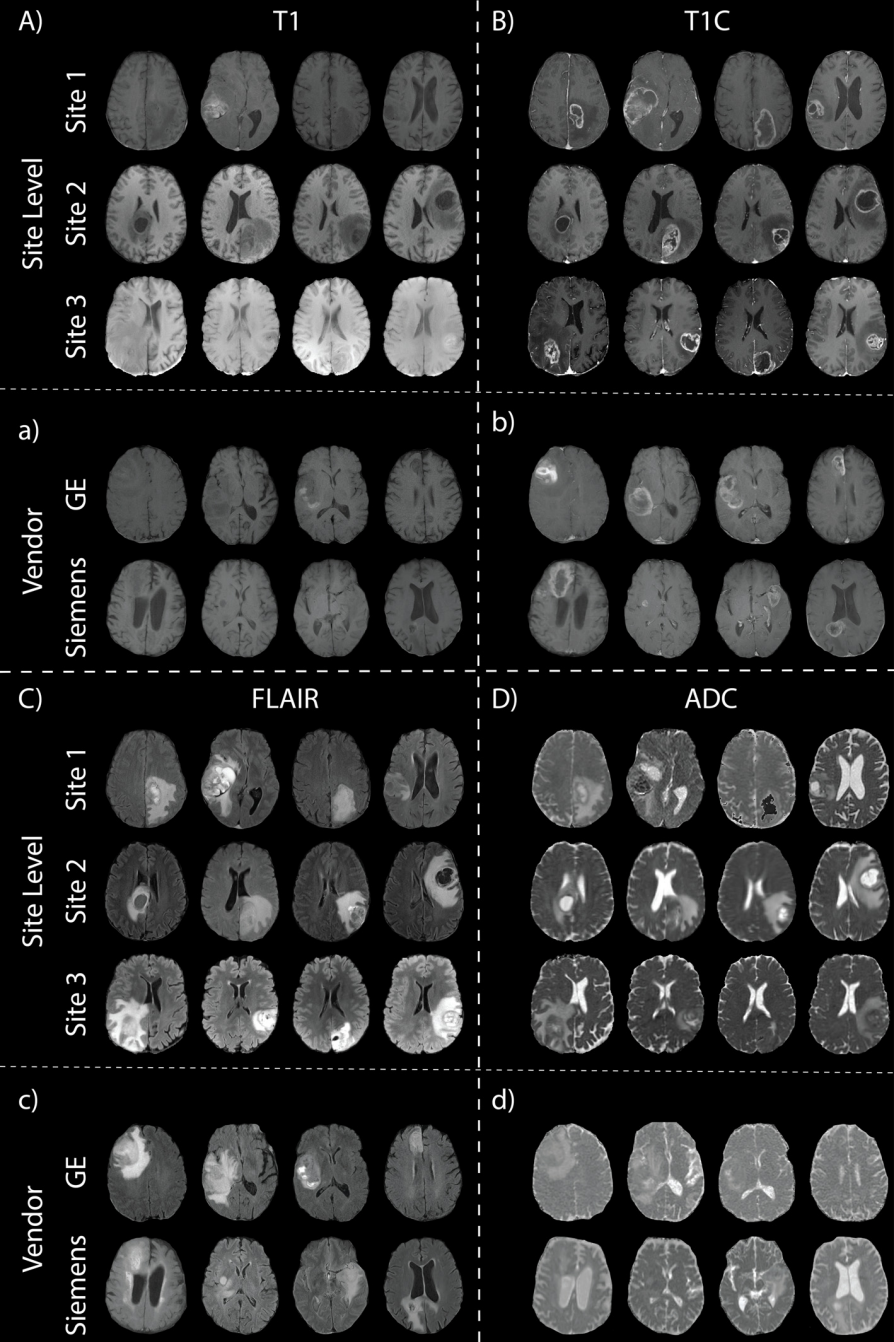
Glioblastoma imaging. T1 **(A)**, T1C **(B)**, FLAIR **(C)**, and ADC **(D)** images for four patients across the three data sites. Additionally, examples of images scanned on the GE and Siemens scanners are shown (**a**, **b**, **c**, **d**, with respect to acquisition).

Written, informed consent was obtained from 52 patients for this cohort, each diagnosed with a glioblastoma in concordance with the 2021 WHO classification standards for brain tumors. Inclusion criteria for this cohort included autopsy confirmed GBM and axial clinical imaging including pre- and post-contrast T1-weighted images (T1, T1C), FLAIR, and DWI 1.5 T (n_1.5T_ = 39, n_3T_ = 13, n_GE_ = 34, n_S_ = 16, n_P_ = 2). Due to the use of clinical imaging, acquisition parameters were not standardized across patients. Axial T1, T1C, FLAIR, and ADC images were selected as the primary acquisitions for this study. ADC maps were calculated using the patient’s clinical DWI. T1, T1C, and ADC images were rigidly aligned to patient’s FLAIR image using SPM12 (https://www.fil.ion.ucl.ac.uk/spm/software/spm12/) (**Table 1** **;** **Figures 2A–D** top rows). Examples of images scanned on the GE and Siemens scanners in **Figure 2** are from this dataset.

#### 2.2.2 Site 2 – UPENN-GBM

Data from this online repository includes MP-MRI for *de novo* GBM patients from the University of Pennsylvania Health System (27, 29). All axial images in this dataset, including T1, T1C, FLAIR, and ADC, were skull-stripped co-registered by an automated computational method (11). A total of 530 patients from this dataset were used after excluding images without all four pre-surgery acquisitions or poor quality (**Table 1** **;** **Figures 2A–D** middle rows).

#### 2.2.3 Site 3 – UCSF-PDGM

Site 3 data come from the publicly available University of California San Francisco Preoperative Diffuse Glioma MRI (UCSF-PDGM) dataset (27, 30). This dataset includes 501 subjects with histopathologically-proven diffuse gliomas who were imaged with a preoperative MRI using a 3T GE Discovery 750. Each image contrast was registered to the FLAIR image (1 mm isotropic resolution) using automated non-linear registration (Advanced Normalization Tools). Resampled co-registered data were then skull stripped using a publicly available deep-learning algorithm (31, 32) **Table 1** **;** **Figures 2A–D** bottom rows). Though a total of 501 adult patients with pathologically confirmed grade II-IV diffuse gliomas were collected for this database, only the 374 patients with confirmed GBM were used.

### 2.3 Breast cancer cohort

**Figure 3:**
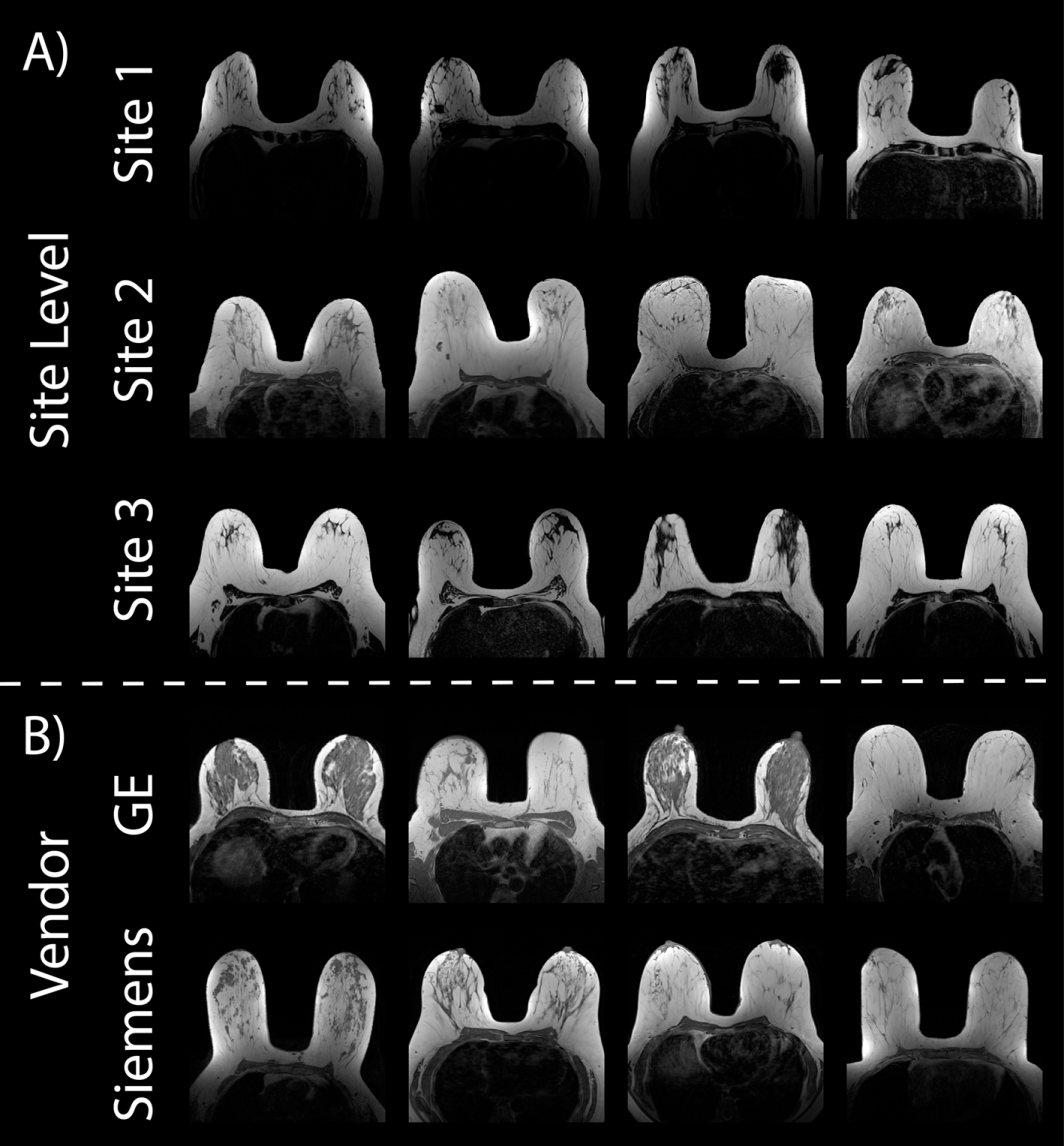
Breast cancer imaging. Example Axial T1 non-fat suppressed images from the three online datasets used in this analysis **(A)**. Vendor-level demonstrations of images **(B)** scanned on the GE (top) and Siemens scanner (bottom) are from Site 2.

All datasets used for our breast imaging analyses were available online (https://cancerimagingarchive.net) (27) and analysis was performed on non-fat suppressed T1 images (T1nFS) (**Figure 3**).

#### 2.3.1 Site 1 – ACRIN 6698

The ACRIN trial 6698, organized by the American College of Radiology Imaging Network, was a multi-institutional research project (33, 34). Its purpose was to determine the efficacy of quantitative DWI in measuring the response of breast cancer to neoadjuvant chemotherapy (NAC). A total 406 women with invasive breast cancer were prospectively enrolled to ACRIN 6698 at ten institutions between August 2012 to January 2015. However, after applying our exclusion criteria described previously in *2.3. Breast Cancer Cohort*, only 68 patients’ images were assessed. All patients underwent breast MRI at 4 timepoints over the course of NAC, though only the pre-treatment images are analyzed in this study. MR imaging was performed on a 1.5T GE scanner using a dedicated breast radiofrequency coil. Detailed MRI protocol parameter specifications can be found on https://cancerimagingarchive.net/ (35).

#### 2.3.2 Site 2 – Duke-Breast-Cancer-MRI

This breast cancer cohort was downloaded from the publicly available MRI dataset (36). The Duke-Breast-Cancer-MRI dataset contains 922 female patients recruited between 2000 and 2014, however, only 351 patients were included in our analyses due to availability of T1nFS images and image quality. Because of annotation constraints described below, a random selection of 100 patients were chosen from the eligible patients for this analysis. As with our local GBM cohort, clinical imaging was provided in the dataset, thus acquisition parameters were not standardized across patients (n_1.5T_ = 49, n_3T_ = 51, n_GE_ = 54, n_S_= 46) (**Figures 3A**, middle; **Figure 3B**).

#### 2.3.3 Site 3 – ISPY2

I-SPY 2 (Investigation of Serial Studies to Predict Your Therapeutic Response with Imaging And moLecular analysis 2) is an ongoing, multi-center study. Its objective is to swiftly assess the effectiveness of novel treatments for breast cancer within the context of NAC (37). Adult women diagnosed with locally advanced breast cancer (tumor size ≥2.5 cm) without distant metastasis recruited between 2010 and 2016 were analyzed for this study. Breast MRI data was acquired prospectively at over 22 clinical centers using a standardized image acquisition protocol. Patients underwent 4 MRI exams before and during NAC, though only the first scan was assessed in the current study. This is a comprehensive, highly curated imaging data set with histopathologic outcome that can be used to develop, test, and compare imaging metrics and prediction models for breast cancer response to treatment. A total of 719 patients were included in this dataset, however, only 68 were assessed after applying the exclusion criteria. MR imaging was performed on a 1.5T GE scanner. All required imaging was performed axially with full bilateral coverage (38).

### 2.4 MRI normalization

Multiple normalization methods were used for each of the three tissue types. Tissue and regions of interest (ROIs) were defined for each tissue type using AFNI (Analysis of Functional NeuroImages, http://afni.nimh.nih.gov/) (39). Prostate masks were manually drawn created on each slice of the patient’s T2-weighted image (T2WI). Brain imaging masks were segmented using SPM12, defined as the combination of the white and gray matter masks. Breast masks were manually drawn on MR images using ITK-Snap. Due to the size of each patient’s imaging, only the center 15 slices were annotated. These tissue masks were used to create the following normalized images for each patient: (1) unnormalized, the (2) standard deviation and (3) z-score of intensity within an individual patient’s tissue mask, (4) min-max, and (5) scaled. All proposed normalization methods were performed at the individual patient level to account for individual variability, preserve biological differences, avoid group-level artifacts and ensure comparability across cohorts whilst maintaining statistical independence. Min-max normalization was defined as the voxelwise subtraction of the minimum intensity value divided by the maximum intensity minus the minimum (Equation 1).


(1) $$\begin{array}{c} normalized\;image=\frac{voxelwise\;intensity-minimum\;intensity}{maximum-minimum\;intensities}\; \end{array}$$

Similarly, the “scaled” normalization was defined as the voxelwise intensity divided by the maximum intensity, scaling all images between 0-1.

**Figure 4:**
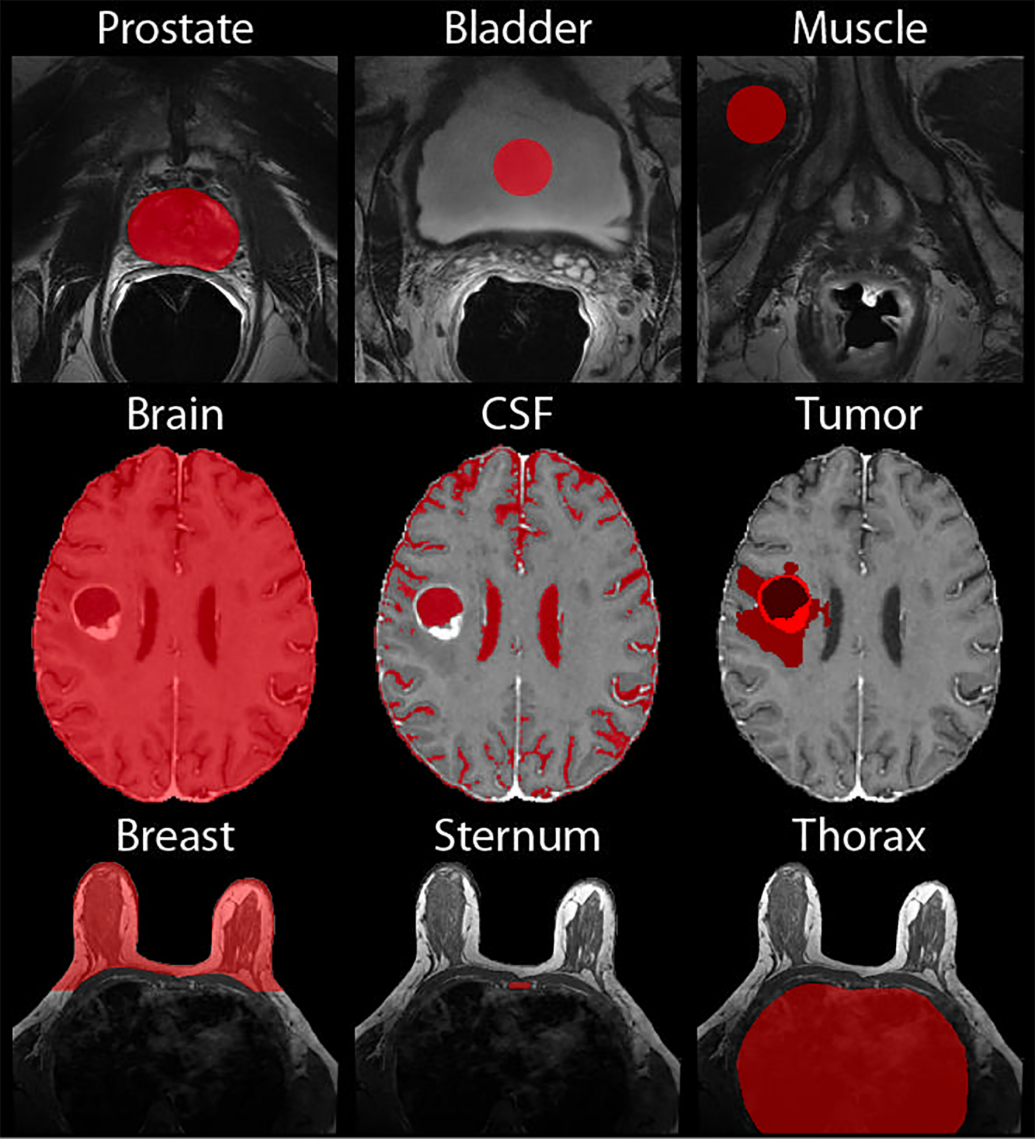
Normalization Masks. Demonstrations of the masks used for normalization of the prostate (top), brain (middle), and breast (bottom).

Two additional ROI-based normalization methods were additionally tested. For prostate images, 10-voxel radius circular ROIs were defined on one slice of the patient’s T2WI within the bladder and levator ani muscle. Corresponding masks were created on the T2WI for patients who had an additional scan done post-endorectal coil removal. For brain images, cerebral spinal fluid (CSF) masks were created by thresholding the ADC for the high diffusion areas, as this is an indicator of fluid. Additionally, a tumor mask was created manually (for Site 1) or using a brain tumor segmentation (BraTs) model, as included in the online data repositories (Sites 2 and 3). These tumor ROIs were defined as the entire tumor region encompassing FLAIR hyperintensity, contrast enhancement, and the necrotic core. Finally, for the breast images, a mask of the sternum was drawn on the axial images, verifying location using the sagittal and coronal images, and the thorax, avoiding any additional tissue. The mean intensity within these ROIs was used for voxelwise normalization. Demonstrations of these masks can be found in **Figure 4**.

### 2.5 Radiomic feature calculation

Radiomic features were calculated across each image using Matlab’s *radiomics* function which calculates a total of 197 features. These include 136 texture features (i.e., 50 gray level co-occurrence matrix (GLCM), 16 gray level dependence zone matrix (GLDZM), 32 gray level run length matrix (GLRLM), 16 gray level size zone matrix (GLSZM), 17 neighboring gray level dependence matrix (NGLDM), and 5 neighboring gray tone difference matrix (NGTDM)), and 61 intensity features (i.e., 18 Intensity Based Statistics, 23 Intensity Histogram, 18 Intensity Volume Histogram, and 2 Local Intensity). All available radiomic features were extracted for analysis to determine if intensity, and/or texture features are affected by normalization techniques.

### 2.6 Statistical analysis

Following normalization, four moments of distribution across MR image intensity (i.e., mean, variance, skewness, and kurtosis), as well as radiomic features, were calculated across patients. Intensity distributions were compared across sites, MR vendors, magnetic field strength (i.e., 1.5T v 3T), and prostate ERC usage using a two one-sided (TOST) test, a test of equivalence that is based on the classical t-test (40). While the TOST test requires both one-sided tests to be statistically significant (i.e., < 0.05), all results described below use the highest p-value for each test.

## 3 Results

**Figure 5:**
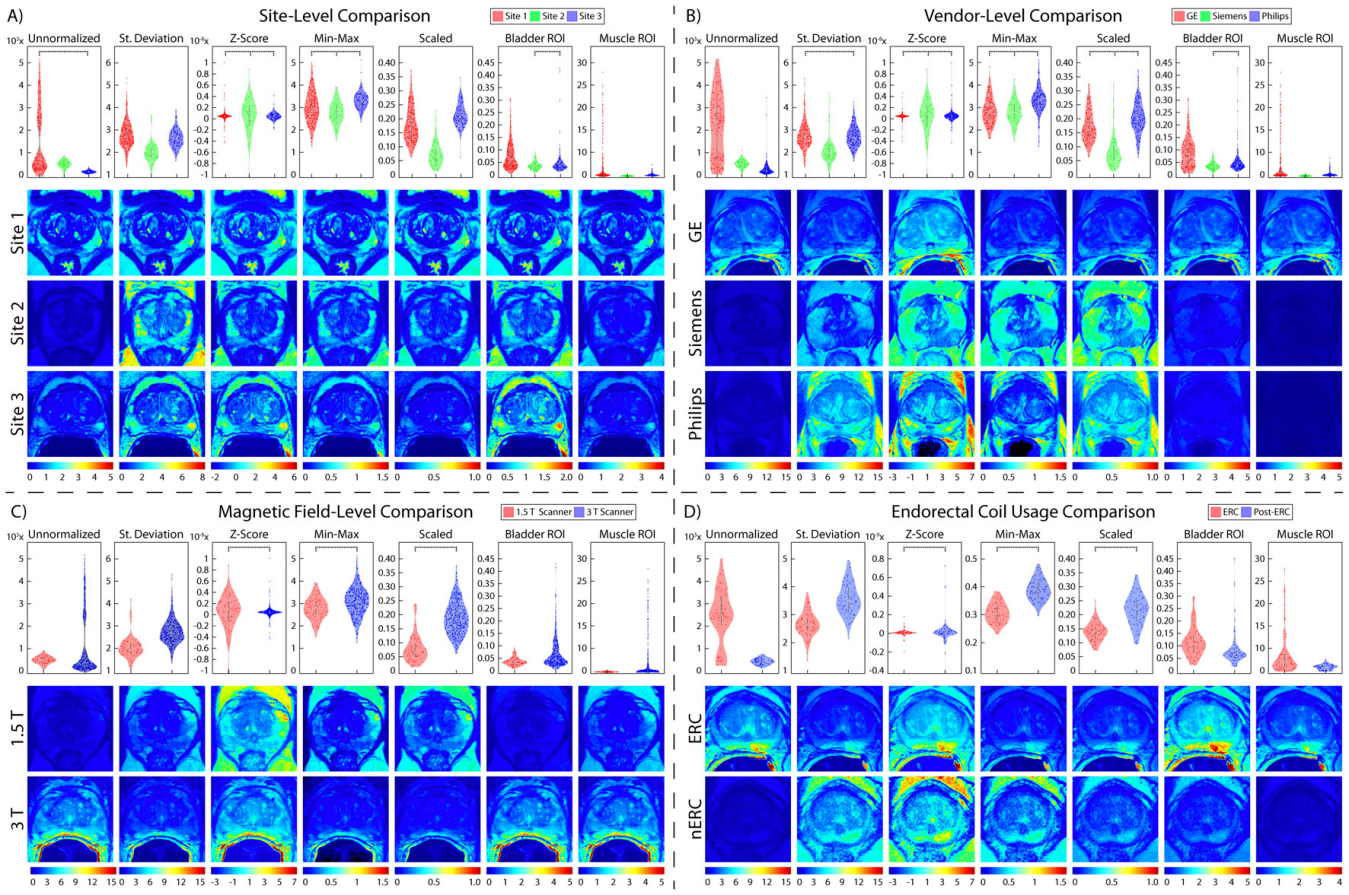
Prostate normalization results. Mean intensity distributions calculated across all normalization comparisons. In each section, mean intensity distribution violin plots are on the top and examples of one patient per comparison are on the bottom. The scales used for the intensity distribution plots as well as the color scale in the visual representations are unique to each tested method. This highlights the differences not only across vendors and ERC usage, but also how different results from each normalization method can be. Pairs of images (i.e., sites, vendors, ERC usage, and magnetic field strength) are displayed on the same scale to compare intensity distributions within each normalization method. **(A)** Site-level normalizations between Site 1 (red, top), Site 2 (green, middle), and Site 3 (blue, bottom). **(B)** Vendor-level normalizations between GE (red, top), Siemens (green, middle), and Philips (blue, bottom). **(C)** Magnetic field strength between 1.5 T (red, top) and 3 T (blue, bottom). **(D)** ERC usage between ERC (red, top) and nERC (blue, bottom).

**Figure 6:**
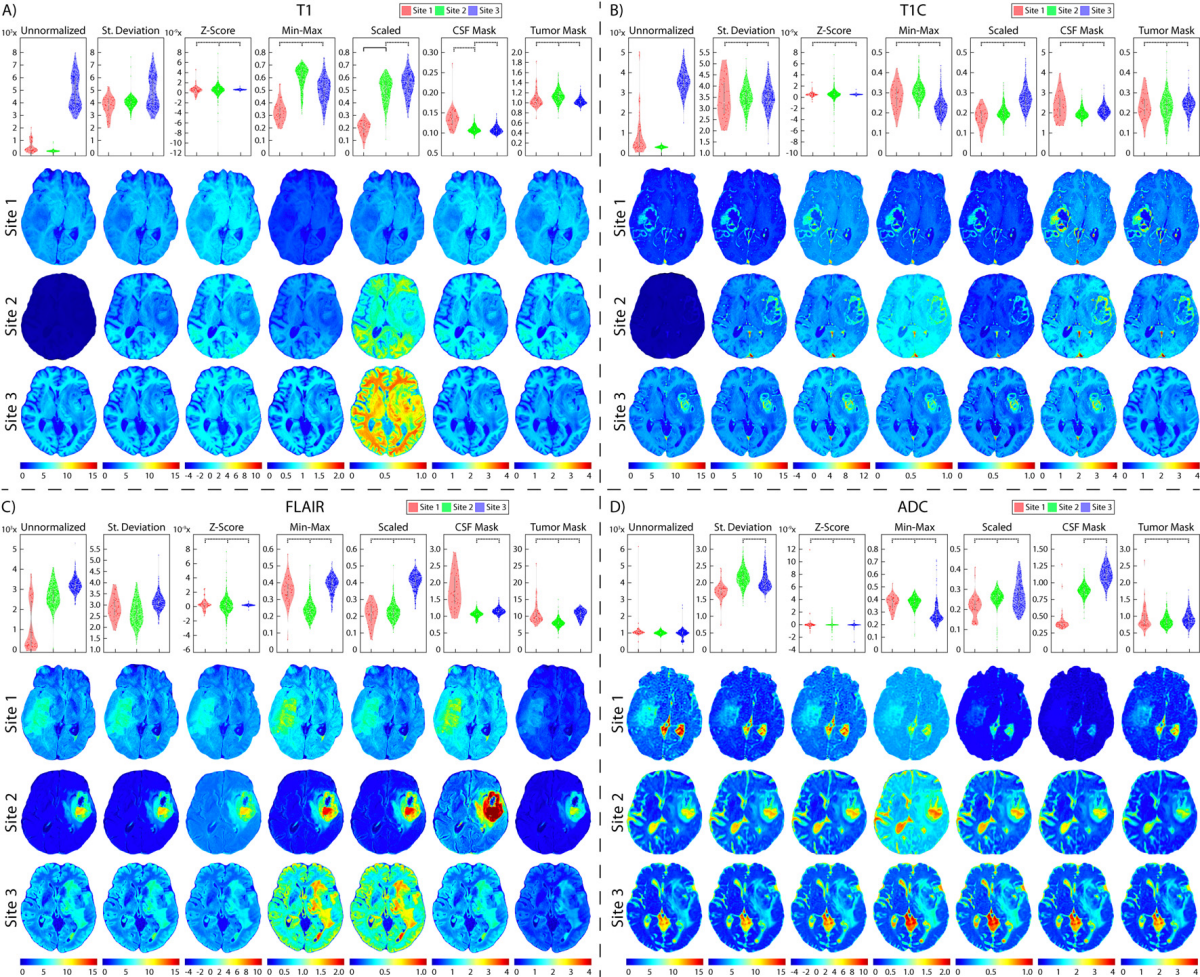
Brain site-level normalization results. Mean intensity distributions calculated across all site-level normalization comparisons in **(A)** T1, **(B)** T1C, **(C)** FLAIR, and **(D)** ADC. In each section, mean intensity distribution plots are on the top and examples of one patient per comparison are on the bottom (Site 1: red, top; Site 2: green, middle; Site 3: blue, bottom). The unnormalized brains especially highlight the differences in intensities between sites, with Site 2 having higher intensities than Site 1.

**Figure 7:**
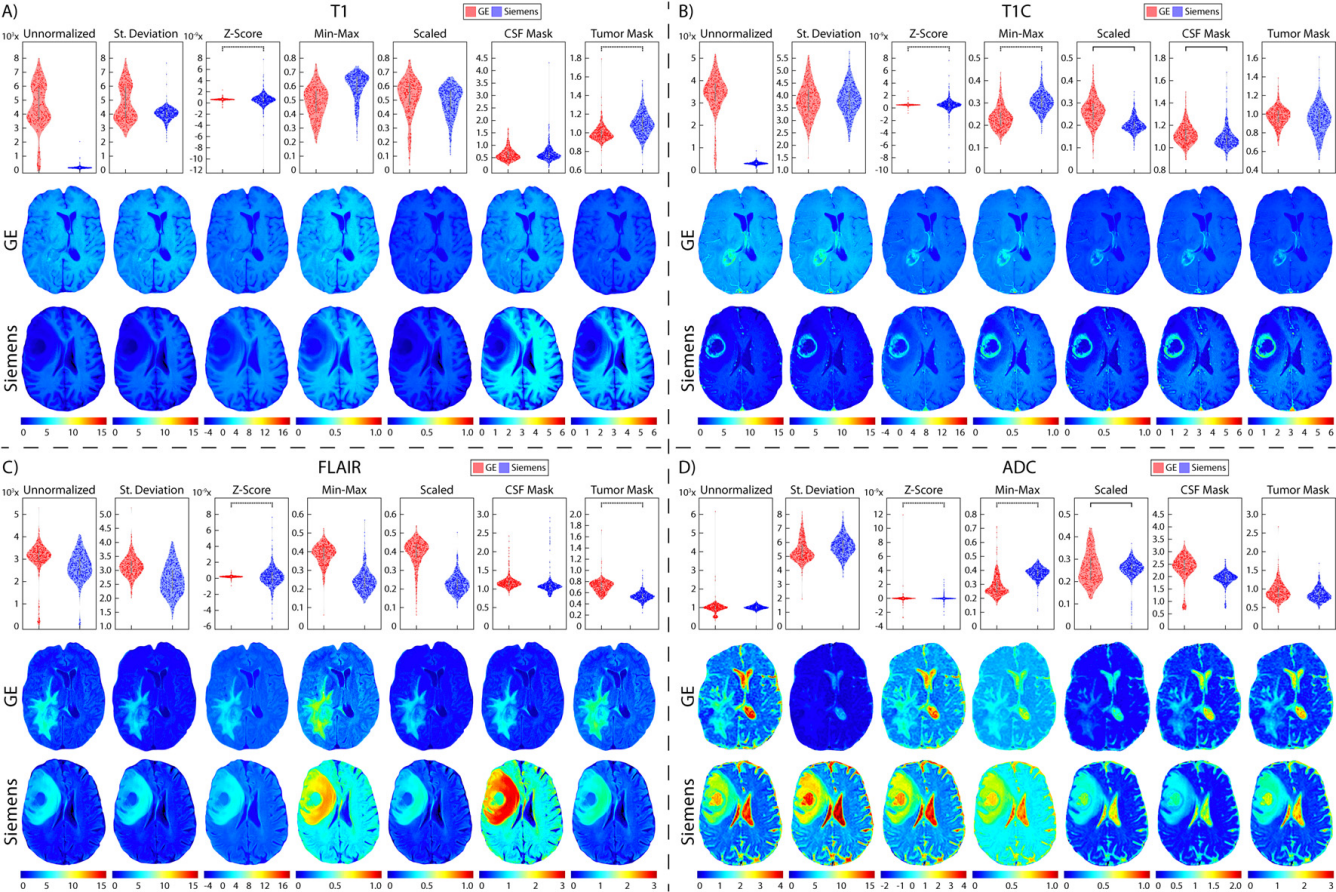
Brain vendor normalization results. Mean intensity distributions calculated across vendor normalization comparisons in **(A)** T1, **(B)** T1C, **(C)** FLAIR, and **(D)** ADC with GE (red, top), and Siemens (blue, middle).

**Figure 8:**
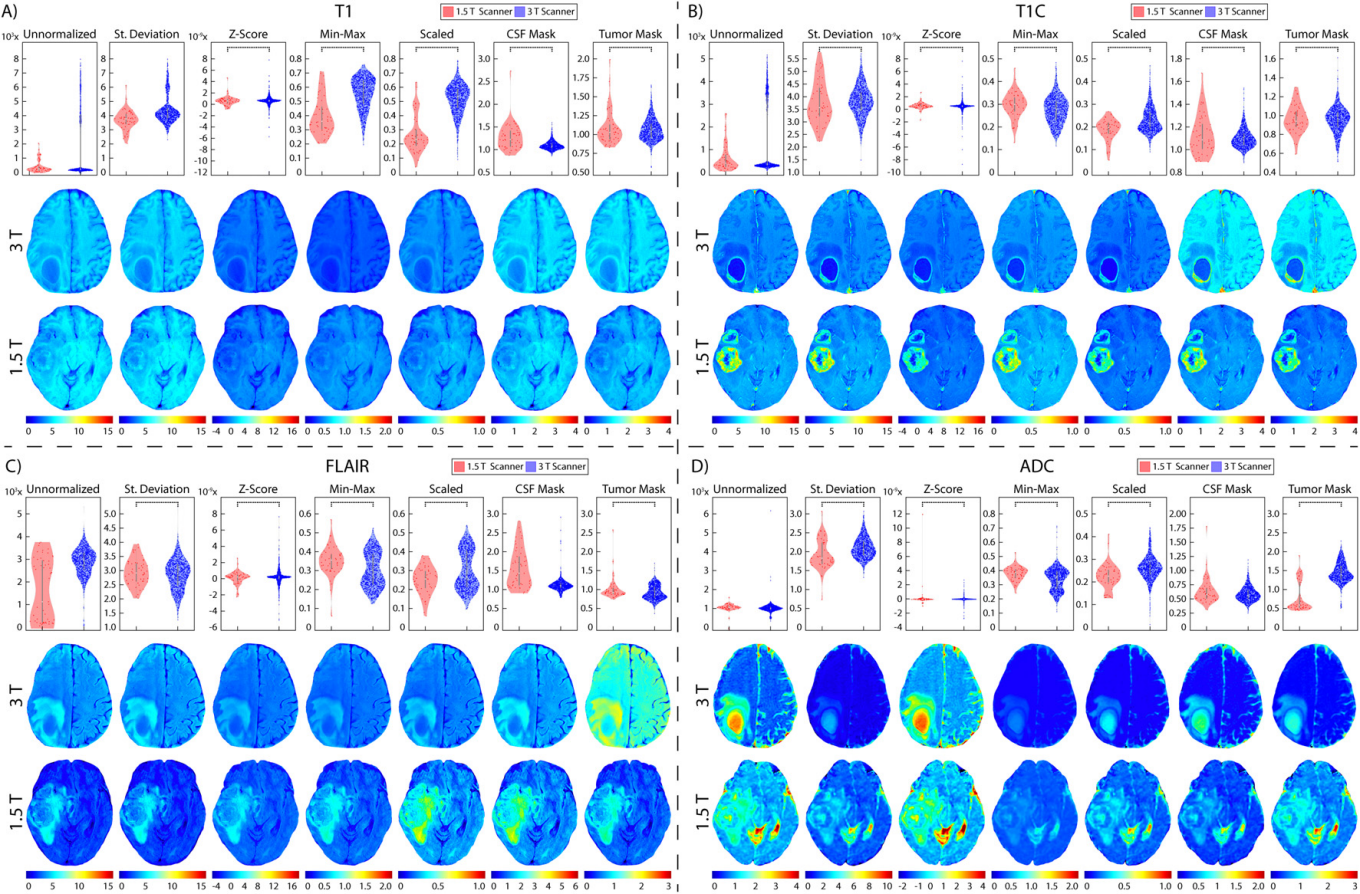
Brain magnetic field normalization results. Mean intensity distributions calculated across magnetic field strength normalization comparisons in **(A)** T1, **(B)** T1C, **(C)** FLAIR, and **(D)** ADC with 1.5 T (red, top), and 3 T (blue, middle).

**Figure 9:**
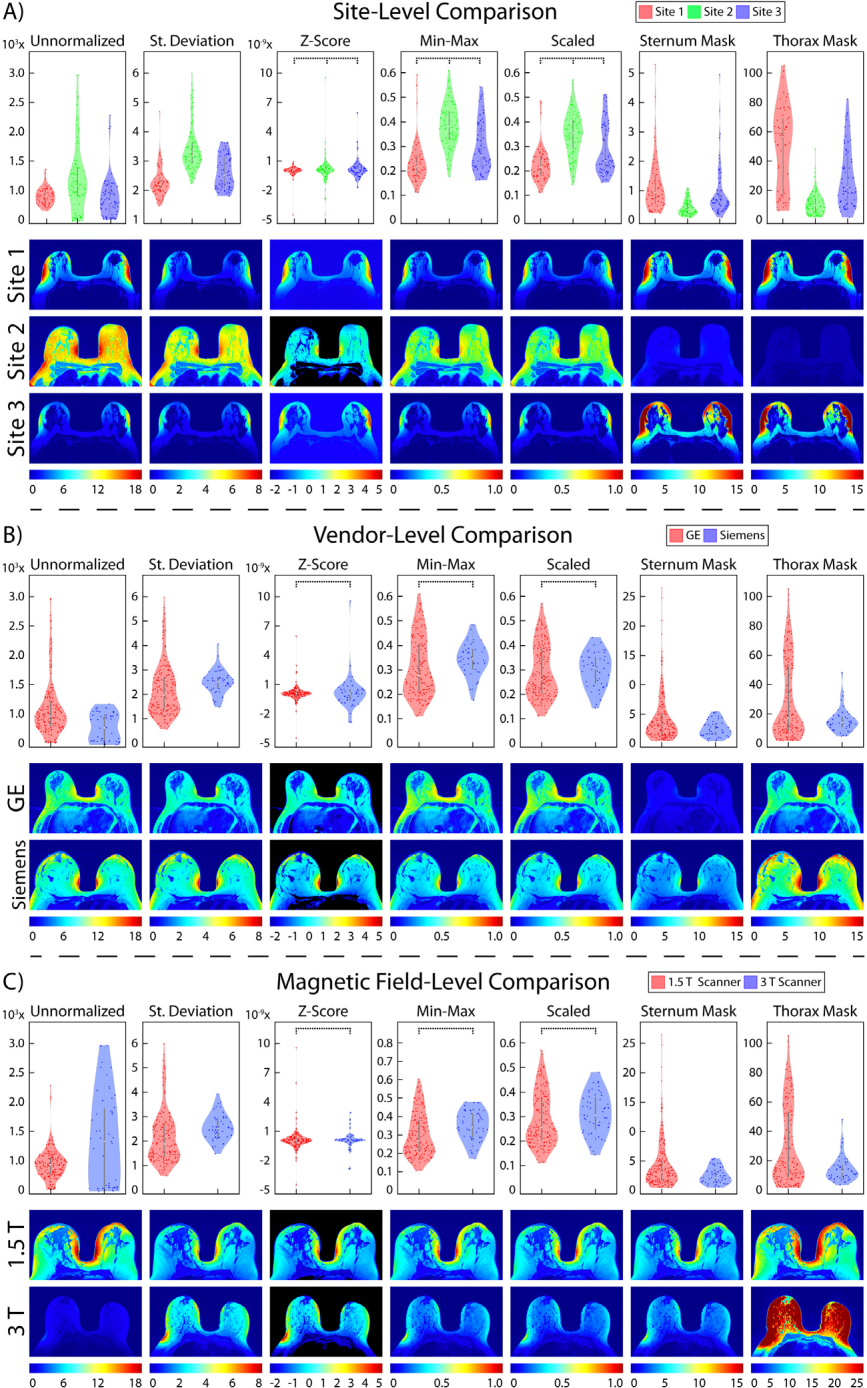
Breast normalization results. Mean intensity distributions calculated across all normalization comparisons. In each section, mean intensity distribution plots are on the top and examples of one patient per comparison are on the bottom. **(A)** Site-level normalizations between Site 1 (red, top), Site 2 (green, middle), and Site 3 (blue, bottom). **(B)** Vendor-level normalizations between GE (red, top), Siemens (blue, bottom). **(C)** Magnetic field strength between 1.5 T (red, top) and 3 T (blue, bottom)

All intensity normalization methods found differing results across the three tissue types, as detailed in the following subsections; however, no kurtosis distribution across any normalization method or comparison were equivalent. **Tables 2**–**7** and **Figures 5**–**9** below present mean intensity values only. The other three moments of distribution results are shown in **Supplementary Tables S1-6**, though they are described below.

**Table 2:** Mean MRI intensity for the seven prostate normalization methods across each intensity comparison.

Normalization Method	Comparison	Pooled St. Deviation	p-value
Unnormalized	Site 1 v Site 2	1170.15	1
	Site 1 v Site 3	1076.48	1
	Site 2 v Site 3	95.78	1
Standard Deviation	Site 1 v Site 2	0.51	1
	Site 1 v Site 3	0.48	<0.001
	Site 2 v Site 3	0.38	1
Z-Score	Site 1 v Site 2	0	<0.001
	Site 1 v Site 3	0	<0.001
	Site 2 v Site 3	0	<0.001
Min-Max	Site 1 v Site 2	0.06	<0.001
	Site 1 v Site 3	0.05	<0.001
	Site 2 v Site 3	0.04	<0.001
Scaled	Site 1 v Site 2	0.05	<0.001
	Site 1 v Site 3	0.05	<0.001
	Site 2 v Site 3	0.04	<0.001
Bladder ROI	Site 1 v Site 2	0.46	0.88
	Site 1 v Site 3	0.49	0.32
	Site 2 v Site 3	0.38	<0.001
Muscle ROI	Site 1 v Site 2	4.34	1
	Site 1 v Site 3	4.01	1
	Site 2 v Site 3	0.35	0.58
Unnormalized	GE v Siemens	1105.9	1
	GE v Philips	893.33	1
	Siemens v Philips	239.93	1
Standard Deviation	GE v Siemens	0.53	1
	GE v Philips	0.48	<0.001
	Siemens v Philips	0.44	1
Z-Score	GE v Siemens	0	<0.001
	GE v Philips	0	<0.001
	Siemens v Philips	0	<0.001
Min-Max	GE v Siemens	0.05	<0.001
	GE v Philips	0.05	<0.001
	Siemens v Philips	0.05	<0.001
Scaled	GE v Siemens	0.05	<0.001
	GE v Philips	0.05	<0.001
	Siemens v Philips	0.05	<0.001
Bladder ROI	GE v Siemens	0.49	1
	GE v Philips	0.47	0.98
	Siemens v Philips	0.35	<0.001
Muscle ROI	GE v Siemens	4.84	1
	GE v Philips	3.86	1
	Siemens v Philips	0.53	0.95
Unnormalized	3 T v 1.5 T	1144.14	1
Standard Deviation	3 T v 1.5 T	0.48	1
Z-Score	3 T v 1.5 T	0	<0.001
Min-Max	3 T v 1.5 T	0.05	<0.001
Scaled	3 T v 1.5 T	0.05	<0.001
Bladder ROI	3 T v 1.5 T	0.48	0.35
Muscle ROI	3 T v 1.5 T	3.84	1
Unnormalized	ERC v nERC	1106.34	0.66
Standard Deviation	ERC v nERC	0.49	<0.001
Z-Score	ERC v nERC	0	<0.001
Min-Max	ERC v nERC	0.04	<0.001
Scaled	ERC v nERC	0.04	<0.001
Bladder ROI	ERC v nERC	0.52	<0.001
Muscle ROI	ERC v nERC	6.24	0.85

ERC, endorectal coil; nERC, post-endorectal coil removal.

**Table 3:** Mean intensity of T1 brain imaging across each of the seven normalization methods.

Normalization Method	Comparison	Pooled St. Deviation	p-value
Unnormalized	Site 1 v Site 2	140.65	1
	Site 1 v Site 3	1102.74	1
	Site 2 v Site 3	750.66	1
Standard Deviation	Site 1 v Site 2	0.43	0.18
	Site 1 v Site 3	1.12	1
	Site 2 v Site 3	0.81	1
Z-Score	Site 1 v Site 2	0	<0.001
	Site 1 v Site 3	0	<0.001
	Site 2 v Site 3	0	<0.001
Min-Max	Site 1 v Site 2	0.08	<0.001
	Site 1 v Site 3	0.09	<0.001
	Site 2 v Site 3	0.08	<0.001
Scaled	Site 1 v Site 2	0.08	0.02
	Site 1 v Site 3	0.08	0.95
	Site 2 v Site 3	0.09	<0.001
CSF Mask	Site 1 v Site 2	0.1	<0.001
	Site 1 v Site 3	0.11	0
	Site 2 v Site 3	0.08	<0.001
Tumor Mask	Site 1 v Site 2	0.11	<0.001
	Site 1 v Site 3	0.08	<0.001
	Site 2 v Site 3	0.09	<0.001
Unnormalized	GE v Siemens	1517.93	1
Standard Deviation	GE v Siemens	1.16	0.67
Z-Score	GE v Siemens	0	<0.001
Min-Max	GE v Siemens	0.1	0.28
Scaled	GE v Siemens	0.12	0.88
CSF Mask	GE v Siemens	0.1	0.24
Tumor Mask	GE v Siemens	0.08	<0.001
Unnormalized	3 T v 1.5 T	2166.08	1
Standard Deviation	3 T v 1.5 T	0.87	1
Z-Score	3 T v 1.5 T	0	<0.001
Min-Max	3 T v 1.5 T	0.1	<0.001
Scaled	3 T v 1.5 T	0.1	<0.001
CSF Mask	3 T v 1.5 T	0.1	<0.001
Tumor Mask	3 T v 1.5 T	0.11	<0.001

CSF, cerebral spinal fluid.

**Table 4:** Mean intensity of T1C brain imaging across each of the seven normalization methods.

Normalization Method	Comparison	Pooled St. Deviation	p-value
Unnormalized	Site 1 v Site 2	286.9	1
	Site 1 v Site 3	587.52	1
	Site 2 v Site 3	335.39	1
Standard Deviation	Site 1 v Site 2	0.49	<0.001
	Site 1 v Site 3	0.56	<0.001
	Site 2 v Site 3	0.48	<0.001
Z-Score	Site 1 v Site 2	0	<0.001
	Site 1 v Site 3	0	<0.001
	Site 2 v Site 3	0	<0.001
Min-Max	Site 1 v Site 2	0.04	<0.001
	Site 1 v Site 3	0.05	<0.001
	Site 2 v Site 3	0.04	<0.001
Scaled	Site 1 v Site 2	0.03	<0.001
	Site 1 v Site 3	0.05	<0.001
	Site 2 v Site 3	0.04	<0.001
CSF Mask	Site 1 v Site 2	0.08	<0.001
	Site 1 v Site 3	0.09	<0.001
	Site 2 v Site 3	0.07	<0.001
Tumor Mask	Site 1 v Site 2	0.14	<0.001
	Site 1 v Site 3	0.09	<0.001
	Site 2 v Site 3	0.12	<0.001
Unnormalized	GE v Siemens	807.01	1
Standard Deviation	GE v Siemens	0.53	0.82
Z-Score	GE v Siemens	0	<0.001
Min-Max	GE v Siemens	0.05	<0.001
Scaled	GE v Siemens	0.05	0.02
CSF Mask	GE v Siemens	0.08	0.03
Tumor Mask	GE v Siemens	0.09	0
Unnormalized	3 T v 1.5 T	1477.97	1
Standard Deviation	3 T v 1.5 T	0.5	<0.001
Z-Score	3 T v 1.5 T	0	<0.001
Min-Max	3 T v 1.5 T	0.05	<0.001
Scaled	3 T v 1.5 T	0.05	<0.001
CSF Mask	3 T v 1.5 T	0.08	<0.001
Tumor Mask	3 T v 1.5 T	0.12	<0.001

CSF, cerebral spinal fluid.

**Table 5:** Mean intensity of FLAIR brain imaging across each of the seven normalization methods.

Normalization Method	Comparison	Pooled St. Deviation	p-value
Unnormalized	Site 1 v Site 2	577.93	1
	Site 1 v Site 3	480.21	1
	Site 2 v Site 3	435.81	1
Standard Deviation	Site 1 v Site 2	0.48	0.12
	Site 1 v Site 3	0.34	0.1
	Site 2 v Site 3	0.42	1
Z-Score	Site 1 v Site 2	0	<0.001
	Site 1 v Site 3	0	<0.001
	Site 2 v Site 3	0	<0.001
Min-Max	Site 1 v Site 2	0.05	<0.001
	Site 1 v Site 3	0.05	<0.001
	Site 2 v Site 3	0.05	<0.001
Scaled	Site 1 v Site 2	0.05	<0.001
	Site 1 v Site 3	0.05	<0.001
	Site 2 v Site 3	0.04	<0.001
CSF Mask	Site 1 v Site 2	0.16	1
	Site 1 v Site 3	0.18	1
	Site 2 v Site 3	0.09	<0.001
Tumor Mask	Site 1 v Site 2	0.09	<0.001
	Site 1 v Site 3	0.1	<0.001
	Site 2 v Site 3	0.07	<0.001
Unnormalized	GE v Siemens	700.74	0.68
Standard Deviation	GE v Siemens	0.33	0.7
Z-Score	GE v Siemens	0	<0.001
Min-Max	GE v Siemens	0.05	0.48
Scaled	GE v Siemens	0.07	0.53
CSF Mask	GE v Siemens	0.16	1
Tumor Mask	GE v Siemens	0.1	0
Unnormalized	3 T v 1.5 T	592.66	1
Standard Deviation	3 T v 1.5 T	0.47	<0.001
Z-Score	3 T v 1.5 T	0	<0.001
Min-Max	3 T v 1.5 T	0.08	<0.001
Scaled	3 T v 1.5 T	0.09	<0.001
CSF Mask	3 T v 1.5 T	0.17	1
Tumor Mask	3 T v 1.5 T	0.12	<0.001

CSF, cerebral spinal fluid.

**Table 6:** Mean intensity of ADC brain imaging across each of the seven normalization methods.

Normalization Method	Comparison	Pooled St. Deviation	p-value
Unnormalized	Site 1 v Site 2	232.75	1
	Site 1 v Site 3	312.75	1
	Site 2 v Site 3	161.89	1
Standard Deviation	Site 1 v Site 2	0.23	0.98
	Site 1 v Site 3	0.25	0.28
	Site 2 v Site 3	0.24	<0.001
Z-Score	Site 1 v Site 2	0	<0.001
	Site 1 v Site 3	0	<0.001
	Site 2 v Site 3	0	<0.001
Min-Max	Site 1 v Site 2	0.04	<0.001
	Site 1 v Site 3	0.06	<0.001
	Site 2 v Site 3	0.05	<0.001
Scaled	Site 1 v Site 2	0.04	<0.001
	Site 1 v Site 3	0.06	<0.001
	Site 2 v Site 3	0.05	<0.001
CSF Mask	Site 1 v Site 2	0.09	1
	Site 1 v Site 3	0.12	1
	Site 2 v Site 3	0.1	<0.001
Tumor Mask	Site 1 v Site 2	0.17	<0.001
	Site 1 v Site 3	0.21	<0.001
	Site 2 v Site 3	0.17	<0.001
Unnormalized	GE v Siemens	314.83	1
Standard Deviation	GE v Siemens	0.27	0.45
Z-Score	GE v Siemens	0	<0.001
Min-Max	GE v Siemens	0.07	<0.001
Scaled	GE v Siemens	0.06	<0.001
CSF Mask	GE v Siemens	0.21	0.93
Tumor Mask	GE v Siemens	0.21	0.15
Unnormalized	3 T v 1.5 T	226.71	0.73
Standard Deviation	3 T v 1.5 T	0.25	<0.001
Z-Score	3 T v 1.5 T	0	<0.001
Min-Max	3 T v 1.5 T	0.07	<0.001
Scaled	3 T v 1.5 T	0.05	<0.001
CSF Mask	3 T v 1.5 T	0.16	1
Tumor Mask	3 T v 1.5 T	0.18	<0.001

CSF, cerebral spinal fluid.

**Table 7:** Mean MRI intensity of each of the seven normalization methods applied to breast imaging.

Normalization Method	Comparison	Pooled St. Deviation	p-value
Unnormalized	Site 1 v Site 2	500.89	1
	Site 1 v Site 3	290.65	0.67
	Site 2 v Site 3	547.86	1
Standard Deviation	Site 1 v Site 2	0.74	1
	Site 1 v Site 3	0.58	0.77
	Site 2 v Site 3	0.75	1
Z-Score	Site 1 v Site 2	0	<0.001
	Site 1 v Site 3	0	<0.001
	Site 2 v Site 3	0	<0.001
Min-Max	Site 1 v Site 2	0.08	<0.001
	Site 1 v Site 3	0.09	<0.001
	Site 2 v Site 3	0.09	<0.001
Scaled	Site 1 v Site 2	0.07	<0.001
	Site 1 v Site 3	0.08	<0.001
	Site 2 v Site 3	0.09	<0.001
Sternum Mask	Site 1 v Site 2	2.8	1
	Site 1 v Site 3	4.04	0.86
	Site 2 v Site 3	2.62	1
Thorax Mask	Site 1 v Site 2	17.06	1
	Site 1 v Site 3	22.3	1
	Site 2 v Site 3	13.03	1
Unnormalized	GE v Siemens	480.05	1
Standard Deviation	GE v Siemens	0.87	0.53
Z-Score	GE v Siemens	0	<0.001
Min-Max	GE v Siemens	0.1	<0.001
Scaled	GE v Siemens	0.09	<0.001
Sternum Mask	GE v Siemens	3.42	0.99
Thorax Mask	GE v Siemens	22.04	1
Unnormalized	3 T v 1.5 T	468.82	1
Standard Deviation	3 T v 1.5 T	0.87	0.74
Z-Score	3 T v 1.5 T	0	<0.001
Min-Max	3 T v 1.5 T	0.1	<0.001
Scaled	3 T v 1.5 T	0.09	<0.001
Sternum Mask	3 T v 1.5 T	3.41	0.99
Thorax Mask	3 T v 1.5 T	21.79	1

### 3.1 Prostate cancer cohort

From our TOST results, we found that across sites and MRI vendors, using the Z-score of masked intensity, Min-Max, and Scaled normalization methods resulted in similar mean and variance intensity distributions (all p < 0.001). Standard deviation normalization likewise found equivalent mean distributions between Site 1-3 and between the GE and Philips vendors, as well as using the bladder ROI between Site 2-3 and the Siemens and Philips vendors (all p < 0.001). Variance distributions were likewise statistically similar using the standard deviation and bladder ROIs across all sites and vendors (both p < 0.001); muscle ROI normalization variance distributions were similar between Site 2-3 and Siemens and Philips vendors (both p < 0.001). Mean and variance distribution comparisons between ERC usage using the standard deviation, Z-Score, Min-Max, Scaled, and bladder ROIs normalization methods resulted in equivalent distributions (all p < 0.001). These results were also observed in magnetic field comparisons, except for the mean intensity after bladder ROI normalization (p = 0.35). All skewness distributions were found to be statistically similar except across any normalization method across Site 2-3 or Siemens and Philips vendors (all others p < 0.001). Mean intensity distribution results for prostate imaging can be found in **Table 2** and **Figure 5**.

### 3.2 Glioblastoma cohort

Two patients were excluded from vendor-level analyses due to being scanned on a Philips scanner and would thus not produce a representative result; these patients were included in the site- and magnetic field-level analyses. In T1 images (**Table 3**, **Figures 6**, **7**, **8A**), we found that at the site-level and across magnetic fields, Z-Score, Min-Max, CSF mask, and tumor mask normalizations produced equivalent mean intensity distributions (all p < 0.001), as well as scaled normalization between Site 1-2 and 2-3 (p = 0.02 and < 0.001, respectively. Skewness across all images between Site 2-3 were found to be significantly similar (al p < 0.001). Across MRI vendors, only Z-score or tumor mask normalized images had similar mean intensity distributions (both p < 0.001). Variance across the normalized images (i.e., all except unnormalized images) for all site, vendor, and magnetic field comparisons were statistically similar (all p < 0.001).

In T1C images (**Table 4**, **Figures 6**, **7**, **8B**), we found that at the site-level and across magnetic fields, mean and variance distributions were statistically similar across all normalized images except for unnormalized (all p < 0.001). Across MRI vendors, all normalization methods besides unnormalized and standard deviation produced equivalent mean intensity distributions (Z-Score, Min-Max, tumor mask p < 0.001; Scaled, CSF mask p < 0.05); however, all but the unnormalized images had equivalent variance distributions (all p < 0.001). No skewness and kurtosis distribution across any image or comparisons was significant.

Across FLAIR images (**Table 5**, **Figures 6**, **7**, **8C**), mean intensity distributions across sites were statistically similar using the Z-score, Min-Max, Scaled, and tumor mask normalizations (all p < 0.001), as well as using the CSF mask between Site 2-3 (p < 0.001). Across MR vendors, only mean intensities using the Z-score and tumor mask normalization were comparable (both p < 0.001). Across magnetic field strengths, all methods besides unnormalized and CSF mask normalization produced equivalent mean distributions (all others p < 0.001). Variance distributions were statistically similar across all sites, vendor, and magnetic field comparison except within unnormalized images (all p < 0.001). As with T1C images, no skewness or kurtosis similarities were found.

Finally, in ADC images (**Table 6**, **Figures 6**, **7**, **8D**), mean intensity across all sites, vendors, and magnetic field strengths were statistically similar using the Z-Score and Min-Max normalizations (all p < 0.001). Standard deviation normalization produced comparable mean intensities across Site 2-3 and magnetic field strength (both p < 0.001). CSF mask normalization additionally had similar mean distributions between Sites 2-3 (both p < 0.001). All mean site- and vendor-level comparisons were statistically similar after Scaled intensity normalization (all p < 0.001), and site- and magnetic field-level comparisons after tumor mask normalization (all p < 0.001). Variance distributions were equivalent for all site- and magnetic field comparisons using all normalization except unnormalized images (all p < 0.001); vendor-level variance distributions were additionally comparable for standard deviation, Z-Score, Min-Max, and Scaled normalizations (all p < 0.001). All skewness distribution comparisons between Site 1-2 and magnetic field strength were statistically similar (Site p < 0.001; Magnetic field p < 0.05).

### 3.3 Breast cancer cohort

In breast imaging, all site, vendor, and magnetic field strength comparisons were significantly equivalent between mean intensity distributions following Z-score, Min-Max, and Scaled normalization, and variance distributions using all normalization methods besides unnormalized images (all p < 0.001) (**Table 7**, **Figure 9**). No skewness or kurtosis similarities were observed.

### 3.4 Radiomic feature analysis

**Figure 10:**
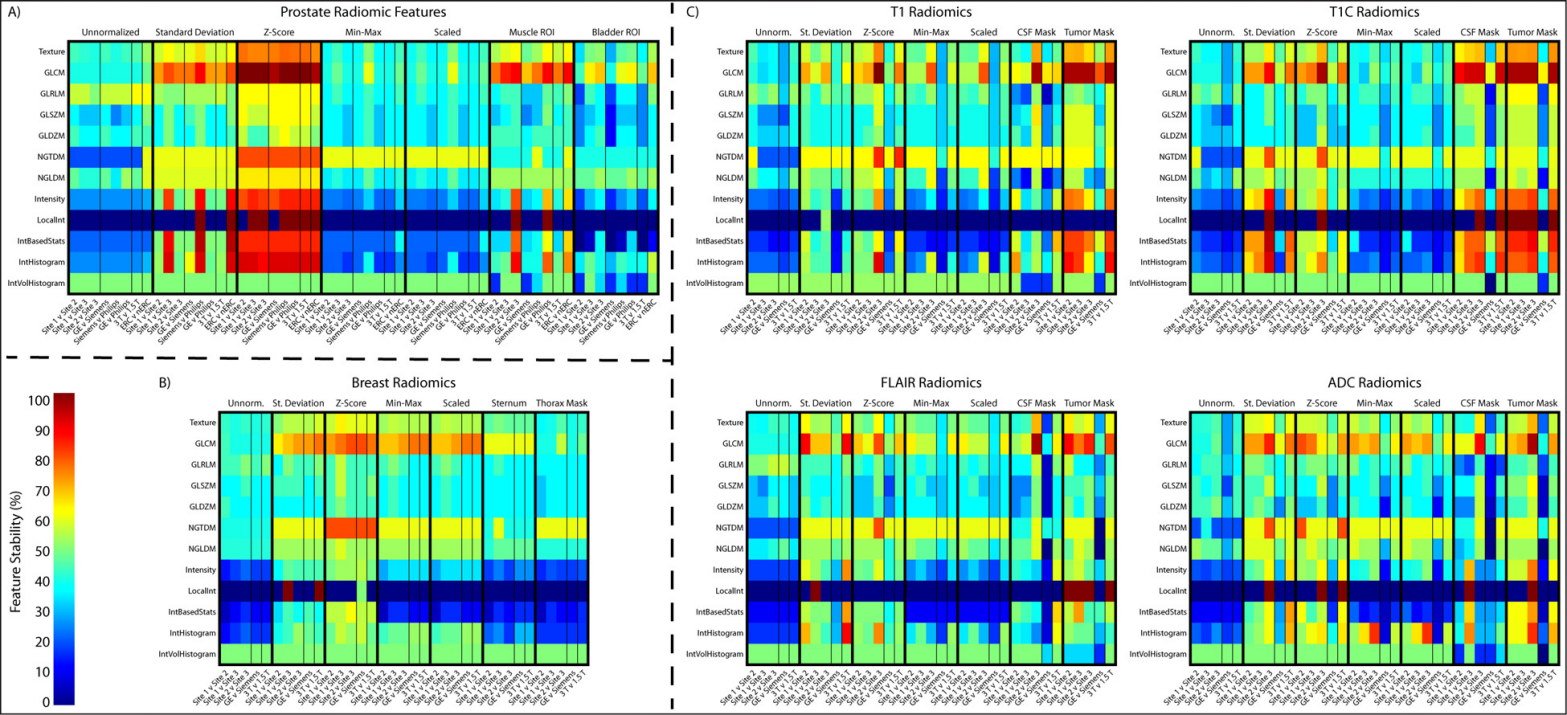
Radiomic feature analysis results across the **(A)** prostate, **(B)** breast, and **(C)** four brain imaging acquisitions. Features are shown as a ratio of number of statistically equivalent results to the number of possible tests per that category.

Similarly to the general intensity analysis, each organ and acquisition had unique results; however, there were general trends across all analyses (**Figure 10**). Standard deviation and Z-score normalization had the highest number and percentage of features that were statistically equal across all acquisitions. Local Intensity had the lowest number of statistically equal features with only 13% being statistically equivalent across acquisitions. GLCM had the highest percent of statistically equal features across all comparisons at 62% statistically comparable. TOST results for each organ can be found in **Supplementary Data Sheets 1-6**. As may be visualized in **Figure 6**, the Site 3 ADC images were not initially scaled consistently with values ranging from millions to 10^-6. Radiomic features were calculated on images scaled to match units. ADC also had the fewest stable radiomic features across every comparison. Prostate radiomic features had the most stability with an average of about 43% intensity, 52% texture, and 50% of all radiomic features. A full breakdown of feature stability across normalized images and by feature class can be found in **Supplementary Table S7**.

## 4 Conclusions

In this study, MP-MRI intensity distributions were assessed to determine the best MR image intensity normalization method for use with quantitative analyses in prostate, glioblastoma, and breast cancer imaging. Two one-sided (TOST) test was used to compare MRI intensities across sites, vendors, and magnetic field strengths used in the three organs, as well using an endorectal coil in prostate imaging. Endorectal coil usage has begun transitioning out of the clinical standard (41–43), thus datasets containing both images with and without an ERC may be impacted by signal intensity differences. Our results suggest that the best normalization for each image acquisition varies; however, in each tested organ and acquisition, the Z-score, Min-Max, and Scaled normalization methods produced comparable images across site, vendors, magnetic field strength, and ERC usage. This can be observed visually using the distributions plots and corresponding maps. Our radiomic feature analyses showed the highest stability of features following standard deviation and Z-scored normalization. These results may indicate that a Z-scored normalization could be applied universally across tissue types with low effect on image intensity and subsequent radiomic analyses.

The standard deviation or Z-score of intensity within each organ was expected to have been skewed due to tumor heterogeneity, including tumor volume and aggressiveness, across patients unrelated to MR vendor differences; however, our results found that normalization using these methods, particularly Z-score, produced the most consistent intensities across vendors and endorectal coil usage. Conversely, ROI-based normalization should have addressed the issue of tumor heterogeneity by using intensities external to the organ; however, we found that ROI-based normalization methods performed poorly in comparison to whole-tissue-based normalization. We also expected the thorax masked breast normalization to perform best among the breast normalization methods, however, it is worthwhile to note that signal heterogeneity exists across breast MR images and few options to test masks external to the breast itself are available. Interestingly, skewness and kurtosis measurements had the least comparisons that were significantly similar following normalization. We had expected those features to capture dataset difference more so than mean and variance, therefore, further research may be warranted to investigate these features with respect to normalization methods.

Intensity normalization is imperative to reduce MRI heterogeneity for quantitative analyses across patients and institutions. While many MRI intensity normalization methods have been established, there is no gold standard method to use, further challenging inter-institutional comparisons. One previous study compared the impact of four normalization methods across T2WI before and after radical external beam radiotherapy (RT) on downstream radiomic feature computations (44). Their methods included (1) unnormalized images, (2) a centered Z-score using mean and standard deviation of image intensity (i.e., Z-score + 3 times the standard deviation), (3) the centered Z-score using the mean and standard deviation of intensity within the bladder, and (4) a histogram-matching approach as proposed by (45). They found that both normalization using the centered Z-score of the image intensity and histogram matching provided the most reproducible radiomic features, whereas ROI-based normalization performed poorly.

In this study, we tested commonly used normalization methods on T2WI across sites, vendors, magnetic field strength, and T2WI across patients scanned with and without an endorectal coil in prostate cancer imaging; T1 non-fat saturated imaging by vendor for breast cancer MRI; and T1, T1C, FLAIR, and ADC in glioblastoma patient imaging to determine the method that produces intensity distributions most similar. Of the methods tested across each tissue type, we found that using Z-scored normalization produces similar intensity distributions across all comparisons, vendors, magnetic field strength, and images with and without an ERC. We additionally calculated 218 radiomic features across images from all normalization methods and found that Z-scored normalization had the highest number of stable features across each comparison. These findings suggest normalization methodology plays a critical role in making inter- and intra-patient MP-MRI-based comparisons.

### 4.1 Limitations

One limitation of this study is the relatively small patient cohort compared to previous MP-MRI analyses for both the prostate and glioblastoma cohorts. Additionally, only two MR vendors were compared across images for glioblastoma and breast, and significantly fewer prostate patients imaged on the Philips scanner. This limited representation could lead to less reliable intensity distributions compared to a larger, more diverse cohort. Furthermore, using clinical imaging acquisitions introduced variability due to non-standardized acquisition parameters which may have differing results when controlling for factors such as field strength. Similarly, image quality was not assessed in this study and should therefore be a topic of future research. Imaging phantoms or repeated scans across multiple vendors may provide more precise intensity distribution estimates, as tissue variability between patients remains a confounding factor. A diverse dataset with repeated patients scans under controlled conditions would allow for accurate similarity measurements within groups using methods such as agreement tests (e.g., intraclass correlation coefficients (ICC) or Cohen’s kappa), correlation tests (e.g., Pearson or Spearman’s correlation coefficient), or distributional similarity tests (e.g., Kolmogorov-Smirnov or Chi-Square).

Lastly, only a selection of normalization methods was tested in this study. It is important to note that several additional normalization methods exist, as previously discussed, such as histogram matching. Histogram matching is a popular technique used in MRI normalization; however, it was untested in this study as it violates several of Shinohara’s principles and was determined in their study to be “inappropriate for any study of images from multiple subjects.” (5) Though most of our normalization methods comply to Shinohara’s principles, we must acknowledge that our tumor-based normalization method does inherently use the patient’s abnormal pathology as an ROI. Our goal was to use a feature of the MRI that exists across all brain MRIs, as was completed for prostate and breast cancer, however, brain Sites 2 and 3 were previously skull-stripped, removing the skull, ears, and eyes which could have been used as a ROI. Additionally, tumor-based normalization would only be possible on cancer-detecting MRI and would thus be rendered useless for brain MRIs with other pathologies. Future studies should compare additional evaluation metrics and techniques to make precise estimates on the most comprehensive image normalization.

### 4.2 Conclusion

We demonstrate in a cohort of 641 prostate cancer patients, 68 of which had scans with and without the use an endorectal coil, 956 glioblastoma patients, and 236 female breast cancer patients, that a Z-scored intensity normalization provides distributions that are most comparable across sites, MR vendors, magnetic field strength, prostate ERC usage, and radiomic feature stability. Using a normalization method that best distributes intensity across tissues could help improve quantitative assessments of cancer MRI. Future studies should investigate larger populations as well as additional MR vendors to determine how normalization methods affect downstream analyses of multi-parametric MR images.

## Data Availability

The raw data supporting the conclusions of this article will be made available by the authors, without undue reservation.
