# Supplementary material for: Comparison of intensity normalization methods in prostate, brain, and breast cancer multi-parametric magnetic resonance imaging
Source: Front Oncol. 2025 Feb 7;15:1433444. doi: 10.3389/fonc.2025.1433444 (PMC11842255; doi:10.3389/fonc.2025.1433444)
Supplement: Supplementary file 1 [file Table1.docx]

Supplementary Material

# **Supplementary Tables**

**Supplemental Table 1: Prostate MRI.** MRI intensity variance, skewness, and kurtosis measurements for the seven prostate normalization methods across each intensity comparison. *Abbreviations: ERC = endorectal coil; nERC = post-endorectal coil removal*.

| **Normalization Method** | **Comparison** | **Pooled St. Deviation** | **p-value** |
| --- | --- | --- | --- |
| **Intensity Variance** | | | |
| Unnormalized | Site 1 v Site 2 | 822592.46 | 1 |
|  | Site 1 v Site 3 | 757378.87 | 1 |
|  | Site 2 v Site 3 | 24493.88 | 1 |
| Standard Deviation | Site 1 v Site 2 | 0 | <0.001 |
|  | Site 1 v Site 3 | 0 | <0.001 |
|  | Site 2 v Site 3 | 0 | <0.001 |
| Z-Score | Site 1 v Site 2 | 0 | <0.001 |
|  | Site 1 v Site 3 | 0 | <0.001 |
|  | Site 2 v Site 3 | 0 | <0.001 |
| Min-Max | Site 1 v Site 2 | 0.01 | <0.001 |
|  | Site 1 v Site 3 | 0.01 | <0.001 |
|  | Site 2 v Site 3 | 0.01 | <0.001 |
| Scaled | Site 1 v Site 2 | 0 | <0.001 |
|  | Site 1 v Site 3 | 0 | <0.001 |
|  | Site 2 v Site 3 | 0 | <0.001 |
| Bladder ROI | Site 1 v Site 2 | 0.18 | <0.001 |
|  | Site 1 v Site 3 | 0.24 | <0.001 |
|  | Site 2 v Site 3 | 0.24 | <0.001 |
| Muscle ROI | Site 1 v Site 2 | 13.5 | 1 |
|  | Site 1 v Site 3 | 12.43 | 1 |
|  | Site 2 v Site 3 | 0.24 | <0.001 |
| Unnormalized | GE v Siemens | 845374.17 | 1 |
|  | GE v Philips | 676219.94 | 1 |
|  | Siemens v Philips | 121920.47 | 1 |
| Standard Deviation | GE v Siemens | 0 | <0.001 |
|  | GE v Philips | 0 | <0.001 |
|  | Siemens v Philips | 0 | <0.001 |
| Z-Score | GE v Siemens | 0 | <0.001 |
|  | GE v Philips | 0 | <0.001 |
|  | Siemens v Philips | 0 | <0.001 |
| Min-Max | GE v Siemens | 0.01 | <0.001 |
|  | GE v Philips | 0.01 | <0.001 |
|  | Siemens v Philips | 0.01 | <0.001 |
| Scaled | GE v Siemens | 0 | <0.001 |
|  | GE v Philips | 0 | <0.001 |
|  | Siemens v Philips | 0 | <0.001 |
| Bladder ROI | GE v Siemens | 0.2 | <0.001 |
|  | GE v Philips | 0.23 | <0.001 |
|  | Siemens v Philips | 0.21 | <0.001 |
| Muscle ROI | GE v Siemens | 15.33 | 1 |
|  | GE v Philips | 12.14 | 1 |
|  | Siemens v Philips | 0.4 | <0.001 |
| Unnormalized | 3 T v 1.5 T | 754711.15 | 1 |
| Standard Deviation | 3 T v 1.5 T | 0 | <0.001 |
| Z-Score | 3 T v 1.5 T | 0 | <0.001 |
| Min-Max | 3 T v 1.5 T | 0.01 | <0.001 |
| Scaled | 3 T v 1.5 T | 0 | <0.001 |
| Bladder ROI | 3 T v 1.5 T | 0.22 | <0.001 |
| Muscle ROI | 3 T v 1.5 T | 11.76 | 0.99 |
| Unnormalized | ERC v nERC | 979507.5 | 0.79 |
| Standard Deviation | ERC v nERC | 0 | <0.001 |
| Z-Score | ERC v nERC | 0 | <0.001 |
| Min-Max | ERC v nERC | 0 | <0.001 |
| Scaled | ERC v nERC | 0 | <0.001 |
| Bladder ROI | ERC v nERC | 0.24 | <0.001 |
| Muscle ROI | ERC v nERC | 20.42 | 0.97 |
| **Intensity Skewness** | | | |
| Unnormalized | Site 1 v Site 2 | 0.43 | <0.001 |
|  | Site 1 v Site 3 | 0.41 | <0.001 |
|  | Site 2 v Site 3 | 0.31 | 0.11 |
| Standard Deviation | Site 1 v Site 2 | 0.43 | <0.001 |
|  | Site 1 v Site 3 | 0.41 | <0.001 |
|  | Site 2 v Site 3 | 0.31 | 0.11 |
| Z-Score | Site 1 v Site 2 | 0.43 | <0.001 |
|  | Site 1 v Site 3 | 0.41 | <0.001 |
|  | Site 2 v Site 3 | 0.31 | 0.11 |
| Min-Max | Site 1 v Site 2 | 0.43 | <0.001 |
|  | Site 1 v Site 3 | 0.41 | <0.001 |
|  | Site 2 v Site 3 | 0.31 | 0.11 |
| Scaled | Site 1 v Site 2 | 0.43 | <0.001 |
|  | Site 1 v Site 3 | 0.41 | <0.001 |
|  | Site 2 v Site 3 | 0.31 | 0.11 |
| Bladder ROI | Site 1 v Site 2 | 0.43 | <0.001 |
|  | Site 1 v Site 3 | 0.41 | <0.001 |
|  | Site 2 v Site 3 | 0.31 | 0.11 |
| Muscle ROI | Site 1 v Site 2 | 0.43 | <0.001 |
|  | Site 1 v Site 3 | 0.41 | <0.001 |
|  | Site 2 v Site 3 | 0.31 | 0.11 |
| Unnormalized | GE v Siemens | 0.38 | <0.001 |
|  | GE v Philips | 0.4 | 0 |
|  | Siemens v Philips | 0.4 | 0.06 |
| Standard Deviation | GE v Siemens | 0.38 | <0.001 |
|  | GE v Philips | 0.4 | 0 |
|  | Siemens v Philips | 0.4 | 0.06 |
| Z-Score | GE v Siemens | 0.38 | <0.001 |
|  | GE v Philips | 0.4 | 0 |
|  | Siemens v Philips | 0.4 | 0.06 |
| Min-Max | GE v Siemens | 0.38 | <0.001 |
|  | GE v Philips | 0.4 | 0 |
|  | Siemens v Philips | 0.4 | 0.06 |
| Scaled | GE v Siemens | 0.38 | <0.001 |
|  | GE v Philips | 0.4 | 0 |
|  | Siemens v Philips | 0.4 | 0.06 |
| Bladder ROI | GE v Siemens | 0.38 | <0.001 |
|  | GE v Philips | 0.4 | 0 |
|  | Siemens v Philips | 0.4 | 0.06 |
| Muscle ROI | GE v Siemens | 0.38 | <0.001 |
|  | GE v Philips | 0.4 | 0 |
|  | Siemens v Philips | 0.4 | 0.06 |
| Unnormalized | 3 T v 1.5 T | 0.4 | 0 |
| Standard Deviation | 3 T v 1.5 T | 0.4 | 0 |
| Z-Score | 3 T v 1.5 T | 0.4 | 0 |
| Min-Max | 3 T v 1.5 T | 0.4 | 0 |
| Scaled | 3 T v 1.5 T | 0.4 | 0 |
| Bladder ROI | 3 T v 1.5 T | 0.4 | 0 |
| Muscle ROI | 3 T v 1.5 T | 0.4 | 0 |
| Unnormalized | ERC v nERC | 0.32 | <0.001 |
| Standard Deviation | ERC v nERC | 0.32 | <0.001 |
| Z-Score | ERC v nERC | 0.32 | <0.001 |
| Min-Max | ERC v nERC | 0.32 | <0.001 |
| Scaled | ERC v nERC | 0.32 | <0.001 |
| Bladder ROI | ERC v nERC | 0.32 | <0.001 |
| Muscle ROI | ERC v nERC | 0.32 | <0.001 |
| **Intensity Kurtosis** | | | |
| Unnormalized | Site 1 v Site 2 | 2.18 | 0.48 |
|  | Site 1 v Site 3 | 2.04 | 0.97 |
|  | Site 2 v Site 3 | 1.23 | 0.68 |
| Standard Deviation | Site 1 v Site 2 | 2.18 | 0.48 |
|  | Site 1 v Site 3 | 2.04 | 0.97 |
|  | Site 2 v Site 3 | 1.23 | 0.68 |
| Z-Score | Site 1 v Site 2 | 2.18 | 0.48 |
|  | Site 1 v Site 3 | 2.04 | 0.97 |
|  | Site 2 v Site 3 | 1.23 | 0.68 |
| Min-Max | Site 1 v Site 2 | 2.18 | 0.48 |
|  | Site 1 v Site 3 | 2.04 | 0.97 |
|  | Site 2 v Site 3 | 1.23 | 0.68 |
| Scaled | Site 1 v Site 2 | 2.18 | 0.48 |
|  | Site 1 v Site 3 | 2.04 | 0.97 |
|  | Site 2 v Site 3 | 1.23 | 0.68 |
| Bladder ROI | Site 1 v Site 2 | 2.18 | 0.48 |
|  | Site 1 v Site 3 | 2.04 | 0.97 |
|  | Site 2 v Site 3 | 1.23 | 0.68 |
| Muscle ROI | Site 1 v Site 2 | 2.18 | 0.48 |
|  | Site 1 v Site 3 | 2.04 | 0.97 |
|  | Site 2 v Site 3 | 1.23 | 0.68 |
| Unnormalized | GE v Siemens | 1.63 | 0.69 |
|  | GE v Philips | 2.05 | 0.89 |
|  | Siemens v Philips | 2.12 | 0.24 |
| Standard Deviation | GE v Siemens | 1.63 | 0.69 |
|  | GE v Philips | 2.05 | 0.89 |
|  | Siemens v Philips | 2.12 | 0.24 |
| Z-Score | GE v Siemens | 1.63 | 0.69 |
|  | GE v Philips | 2.05 | 0.89 |
|  | Siemens v Philips | 2.12 | 0.24 |
| Min-Max | GE v Siemens | 1.63 | 0.69 |
|  | GE v Philips | 2.05 | 0.89 |
|  | Siemens v Philips | 2.12 | 0.24 |
| Scaled | GE v Siemens | 1.63 | 0.69 |
|  | GE v Philips | 2.05 | 0.89 |
|  | Siemens v Philips | 2.12 | 0.24 |
| Bladder ROI | GE v Siemens | 1.63 | 0.69 |
|  | GE v Philips | 2.05 | 0.89 |
|  | Siemens v Philips | 2.12 | 0.24 |
| Muscle ROI | GE v Siemens | 1.63 | 0.69 |
|  | GE v Philips | 2.05 | 0.89 |
|  | Siemens v Philips | 2.12 | 0.24 |
| Unnormalized | 3 T v 1.5 T | 1.98 | 0.16 |
| Standard Deviation | 3 T v 1.5 T | 1.98 | 0.16 |
| Z-Score | 3 T v 1.5 T | 1.98 | 0.16 |
| Min-Max | 3 T v 1.5 T | 1.98 | 0.16 |
| Scaled | 3 T v 1.5 T | 1.98 | 0.16 |
| Bladder ROI | 3 T v 1.5 T | 1.98 | 0.16 |
| Muscle ROI | 3 T v 1.5 T | 1.98 | 0.16 |
| Unnormalized | ERC v nERC | 1.41 | 0.08 |
| Standard Deviation | ERC v nERC | 1.41 | 0.08 |
| Z-Score | ERC v nERC | 1.41 | 0.08 |
| Min-Max | ERC v nERC | 1.41 | 0.08 |
| Scaled | ERC v nERC | 1.41 | 0.08 |
| Bladder ROI | ERC v nERC | 1.41 | 0.08 |
| Muscle ROI | ERC v nERC | 1.41 | 0.08 |

**Supplemental Table 2: Brain T1.** MRI intensity variance, skewness, and kurtosis measurements for the seven brain normalization methods across each intensity comparison. *Abbreviations: CSF = cerebral spinal fluid*.

| **Normalization Method** | **Comparison** | **Pooled St. Deviation** | **p-value** |
| --- | --- | --- | --- |
| **Intensity Variance** | | | |
| Unnormalized | Site 1 v Site 2 | 46496.51 | 1 |
|  | Site 1 v Site 3 | 54308.95 | 1 |
|  | Site 2 v Site 3 | 1925.68 | 1 |
| Standard Deviation | Site 1 v Site 2 | 0 | <0.001 |
|  | Site 1 v Site 3 | 0 | <0.001 |
|  | Site 2 v Site 3 | 0 | <0.001 |
| Z-Score | Site 1 v Site 2 | 0 | <0.001 |
|  | Site 1 v Site 3 | 0 | <0.001 |
|  | Site 2 v Site 3 | 0 | <0.001 |
| Min-Max | Site 1 v Site 2 | 0 | <0.001 |
|  | Site 1 v Site 3 | 0.01 | <0.001 |
|  | Site 2 v Site 3 | 0.01 | <0.001 |
| Scaled | Site 1 v Site 2 | 0.01 | <0.001 |
|  | Site 1 v Site 3 | 0.01 | <0.001 |
|  | Site 2 v Site 3 | 0.01 | <0.001 |
| CSF Mask | Site 1 v Site 2 | 0.07 | <0.001 |
|  | Site 1 v Site 3 | 0.08 | <0.001 |
|  | Site 2 v Site 3 | 0.03 | <0.001 |
| Tumor Mask | Site 1 v Site 2 | 0.03 | <0.001 |
|  | Site 1 v Site 3 | 0.04 | <0.001 |
|  | Site 2 v Site 3 | 0.03 | <0.001 |
| Unnormalized | GE v Siemens | 247478.43 | 1 |
| Standard Deviation | GE v Siemens | 0 | <0.001 |
| Z-Score | GE v Siemens | 0 | <0.001 |
| Min-Max | GE v Siemens | 0.01 | <0.001 |
| Scaled | GE v Siemens | 0.01 | <0.001 |
| CSF Mask | GE v Siemens | 0.04 | <0.001 |
| Tumor Mask | GE v Siemens | 0.03 | <0.001 |
| Unnormalized | 3 T v 1.5 T | 476308.93 | 1 |
| Standard Deviation | 3 T v 1.5 T | 0 | <0.001 |
| Z-Score | 3 T v 1.5 T | 0 | <0.001 |
| Min-Max | 3 T v 1.5 T | 0.01 | <0.001 |
| Scaled | 3 T v 1.5 T | 0.01 | <0.001 |
| CSF Mask | 3 T v 1.5 T | 0.06 | <0.001 |
| Tumor Mask | 3 T v 1.5 T | 0.03 | <0.001 |
| **Intensity Skewness** | | | |
| Unnormalized | Site 1 v Site 2 | 0.49 | 1 |
|  | Site 1 v Site 3 | 0.66 | 1 |
|  | Site 2 v Site 3 | 0.38 | <0.001 |
| Standard Deviation | Site 1 v Site 2 | 0.49 | 1 |
|  | Site 1 v Site 3 | 0.66 | 1 |
|  | Site 2 v Site 3 | 0.38 | <0.001 |
| Z-Score | Site 1 v Site 2 | 0.49 | 1 |
|  | Site 1 v Site 3 | 0.66 | 1 |
|  | Site 2 v Site 3 | 0.38 | <0.001 |
| Min-Max | Site 1 v Site 2 | 0.49 | 1 |
|  | Site 1 v Site 3 | 0.66 | 1 |
|  | Site 2 v Site 3 | 0.38 | <0.001 |
| Scaled | Site 1 v Site 2 | 0.49 | 1 |
|  | Site 1 v Site 3 | 0.66 | 1 |
|  | Site 2 v Site 3 | 0.38 | <0.001 |
| CSF Mask | Site 1 v Site 2 | 0.49 | 1 |
|  | Site 1 v Site 3 | 0.66 | 1 |
|  | Site 2 v Site 3 | 0.38 | <0.001 |
| Tumor Mask | Site 1 v Site 2 | 0.49 | 1 |
|  | Site 1 v Site 3 | 0.66 | 1 |
|  | Site 2 v Site 3 | 0.38 | <0.001 |
| Unnormalized | GE v Siemens | 0.59 | 1 |
| Standard Deviation | GE v Siemens | 0.59 | 1 |
| Z-Score | GE v Siemens | 0.59 | 1 |
| Min-Max | GE v Siemens | 0.59 | 1 |
| Scaled | GE v Siemens | 0.59 | 1 |
| CSF Mask | GE v Siemens | 0.59 | 1 |
| Tumor Mask | GE v Siemens | 0.59 | 1 |
| Unnormalized | 3 T v 1.5 T | 0.51 | 1 |
| Standard Deviation | 3 T v 1.5 T | 0.51 | 1 |
| Z-Score | 3 T v 1.5 T | 0.51 | 1 |
| Min-Max | 3 T v 1.5 T | 0.51 | 1 |
| Scaled | 3 T v 1.5 T | 0.51 | 1 |
| CSF Mask | 3 T v 1.5 T | 0.51 | 1 |
| Tumor Mask | 3 T v 1.5 T | 0.51 | 1 |
| **Intensity Kurtosis** | | | |
| Unnormalized | Site 1 v Site 2 | 11.83 | 1 |
|  | Site 1 v Site 3 | 13.91 | 1 |
|  | Site 2 v Site 3 | 1.56 | 0.7 |
| Standard Deviation | Site 1 v Site 2 | 11.83 | 1 |
|  | Site 1 v Site 3 | 13.91 | 1 |
|  | Site 2 v Site 3 | 1.56 | 0.7 |
| Z-Score | Site 1 v Site 2 | 11.83 | 1 |
|  | Site 1 v Site 3 | 13.91 | 1 |
|  | Site 2 v Site 3 | 1.56 | 0.7 |
| Min-Max | Site 1 v Site 2 | 11.83 | 1 |
|  | Site 1 v Site 3 | 13.91 | 1 |
|  | Site 2 v Site 3 | 1.56 | 0.7 |
| Scaled | Site 1 v Site 2 | 11.83 | 1 |
|  | Site 1 v Site 3 | 13.91 | 1 |
|  | Site 2 v Site 3 | 1.56 | 0.7 |
| CSF Mask | Site 1 v Site 2 | 11.83 | 1 |
|  | Site 1 v Site 3 | 13.91 | 1 |
|  | Site 2 v Site 3 | 1.56 | 0.7 |
| Tumor Mask | Site 1 v Site 2 | 11.83 | 1 |
|  | Site 1 v Site 3 | 13.91 | 1 |
|  | Site 2 v Site 3 | 1.56 | 0.7 |
| Unnormalized | GE v Siemens | 2.46 | 1 |
| Standard Deviation | GE v Siemens | 2.46 | 1 |
| Z-Score | GE v Siemens | 2.46 | 1 |
| Min-Max | GE v Siemens | 2.46 | 1 |
| Scaled | GE v Siemens | 2.46 | 1 |
| CSF Mask | GE v Siemens | 2.46 | 1 |
| Tumor Mask | GE v Siemens | 2.46 | 1 |
| Unnormalized | 3 T v 1.5 T | 9.37 | 1 |
| Standard Deviation | 3 T v 1.5 T | 9.37 | 1 |
| Z-Score | 3 T v 1.5 T | 9.37 | 1 |
| Min-Max | 3 T v 1.5 T | 9.37 | 1 |
| Scaled | 3 T v 1.5 T | 9.37 | 1 |
| CSF Mask | 3 T v 1.5 T | 9.37 | 1 |
| Tumor Mask | 3 T v 1.5 T | 9.37 | 1 |

**Supplemental Table 3: Brain T1C.** MRI intensity variance, skewness, and kurtosis measurements for the seven brain normalization methods across each intensity comparison. *Abbreviations: CSF = cerebral spinal fluid*.

| **Normalization Method** | **Comparison** | **Pooled St. Deviation** | **p-value** |
| --- | --- | --- | --- |
| **Intensity Variance** | | | |
| Unnormalized | Site 1 v Site 2 | 140635.22 | 1 |
|  | Site 1 v Site 3 | 164373.78 | 1 |
|  | Site 2 v Site 3 | 4138.39 | 1 |
| Standard Deviation | Site 1 v Site 2 | 0 | <0.001 |
|  | Site 1 v Site 3 | 0 | <0.001 |
|  | Site 2 v Site 3 | 0 | <0.001 |
| Z-Score | Site 1 v Site 2 | 0 | <0.001 |
|  | Site 1 v Site 3 | 0 | <0.001 |
|  | Site 2 v Site 3 | 0 | <0.001 |
| Min-Max | Site 1 v Site 2 | 0 | <0.001 |
|  | Site 1 v Site 3 | 0 | <0.001 |
|  | Site 2 v Site 3 | 0 | <0.001 |
| Scaled | Site 1 v Site 2 | 0 | <0.001 |
|  | Site 1 v Site 3 | 0 | <0.001 |
|  | Site 2 v Site 3 | 0 | <0.001 |
| CSF Mask | Site 1 v Site 2 | 0.05 | <0.001 |
|  | Site 1 v Site 3 | 0.06 | <0.001 |
|  | Site 2 v Site 3 | 0.04 | <0.001 |
| Tumor Mask | Site 1 v Site 2 | 0.03 | <0.001 |
|  | Site 1 v Site 3 | 0.04 | <0.001 |
|  | Site 2 v Site 3 | 0.03 | <0.001 |
| Unnormalized | GE v Siemens | 243038.87 | 1 |
| Standard Deviation | GE v Siemens | 0 | <0.001 |
| Z-Score | GE v Siemens | 0 | <0.001 |
| Min-Max | GE v Siemens | 0 | <0.001 |
| Scaled | GE v Siemens | 0 | <0.001 |
| CSF Mask | GE v Siemens | 0.05 | <0.001 |
| Tumor Mask | GE v Siemens | 0.04 | <0.001 |
| Unnormalized | 3 T v 1.5 T | 481327.57 | 1 |
| Standard Deviation | 3 T v 1.5 T | 0 | <0.001 |
| Z-Score | 3 T v 1.5 T | 0 | <0.001 |
| Min-Max | 3 T v 1.5 T | 0 | <0.001 |
| Scaled | 3 T v 1.5 T | 0 | <0.001 |
| CSF Mask | 3 T v 1.5 T | 0.05 | <0.001 |
| Tumor Mask | 3 T v 1.5 T | 0.03 | <0.001 |
| **Intensity Skewness** | | | |
| Unnormalized | Site 1 v Site 2 | 0.88 | 0.57 |
|  | Site 1 v Site 3 | 1 | 1 |
|  | Site 2 v Site 3 | 0.92 | 1 |
| Standard Deviation | Site 1 v Site 2 | 0.88 | 0.57 |
|  | Site 1 v Site 3 | 1 | 1 |
|  | Site 2 v Site 3 | 0.92 | 1 |
| Z-Score | Site 1 v Site 2 | 0.88 | 0.57 |
|  | Site 1 v Site 3 | 1 | 1 |
|  | Site 2 v Site 3 | 0.92 | 1 |
| Min-Max | Site 1 v Site 2 | 0.88 | 0.57 |
|  | Site 1 v Site 3 | 1 | 1 |
|  | Site 2 v Site 3 | 0.92 | 1 |
| Scaled | Site 1 v Site 2 | 0.88 | 0.57 |
|  | Site 1 v Site 3 | 1 | 1 |
|  | Site 2 v Site 3 | 0.92 | 1 |
| CSF Mask | Site 1 v Site 2 | 0.88 | 0.57 |
|  | Site 1 v Site 3 | 1 | 1 |
|  | Site 2 v Site 3 | 0.92 | 1 |
| Tumor Mask | Site 1 v Site 2 | 0.88 | 0.57 |
|  | Site 1 v Site 3 | 1 | 1 |
|  | Site 2 v Site 3 | 0.92 | 1 |
| Unnormalized | GE v Siemens | 1.04 | 0.86 |
| Standard Deviation | GE v Siemens | 1.04 | 0.86 |
| Z-Score | GE v Siemens | 1.04 | 0.86 |
| Min-Max | GE v Siemens | 1.04 | 0.86 |
| Scaled | GE v Siemens | 1.04 | 0.86 |
| CSF Mask | GE v Siemens | 1.04 | 0.86 |
| Tumor Mask | GE v Siemens | 1.04 | 0.86 |
| Unnormalized | 3 T v 1.5 T | 0.99 | 0.96 |
| Standard Deviation | 3 T v 1.5 T | 0.99 | 0.96 |
| Z-Score | 3 T v 1.5 T | 0.99 | 0.96 |
| Min-Max | 3 T v 1.5 T | 0.99 | 0.96 |
| Scaled | 3 T v 1.5 T | 0.99 | 0.96 |
| CSF Mask | 3 T v 1.5 T | 0.99 | 0.96 |
| Tumor Mask | 3 T v 1.5 T | 0.99 | 0.96 |
| **Intensity Kurtosis** | | | |
| Unnormalized | Site 1 v Site 2 | 8.08 | 0.52 |
|  | Site 1 v Site 3 | 9.63 | 0.97 |
|  | Site 2 v Site 3 | 5.65 | 1 |
| Standard Deviation | Site 1 v Site 2 | 8.08 | 0.52 |
|  | Site 1 v Site 3 | 9.63 | 0.97 |
|  | Site 2 v Site 3 | 5.65 | 1 |
| Z-Score | Site 1 v Site 2 | 8.08 | 0.52 |
|  | Site 1 v Site 3 | 9.63 | 0.97 |
|  | Site 2 v Site 3 | 5.65 | 1 |
| Min-Max | Site 1 v Site 2 | 8.08 | 0.52 |
|  | Site 1 v Site 3 | 9.63 | 0.97 |
|  | Site 2 v Site 3 | 5.65 | 1 |
| Scaled | Site 1 v Site 2 | 8.08 | 0.52 |
|  | Site 1 v Site 3 | 9.63 | 0.97 |
|  | Site 2 v Site 3 | 5.65 | 1 |
| CSF Mask | Site 1 v Site 2 | 8.08 | 0.52 |
|  | Site 1 v Site 3 | 9.63 | 0.97 |
|  | Site 2 v Site 3 | 5.65 | 1 |
| Tumor Mask | Site 1 v Site 2 | 8.08 | 0.52 |
|  | Site 1 v Site 3 | 9.63 | 0.97 |
|  | Site 2 v Site 3 | 5.65 | 1 |
| Unnormalized | GE v Siemens | 6.41 | 1 |
| Standard Deviation | GE v Siemens | 6.41 | 1 |
| Z-Score | GE v Siemens | 6.41 | 1 |
| Min-Max | GE v Siemens | 6.41 | 1 |
| Scaled | GE v Siemens | 6.41 | 1 |
| CSF Mask | GE v Siemens | 6.41 | 1 |
| Tumor Mask | GE v Siemens | 6.41 | 1 |
| Unnormalized | 3 T v 1.5 T | 7.55 | 0.75 |
| Standard Deviation | 3 T v 1.5 T | 7.55 | 0.75 |
| Z-Score | 3 T v 1.5 T | 7.55 | 0.75 |
| Min-Max | 3 T v 1.5 T | 7.55 | 0.75 |
| Scaled | 3 T v 1.5 T | 7.55 | 0.75 |
| CSF Mask | 3 T v 1.5 T | 7.55 | 0.75 |
| Tumor Mask | 3 T v 1.5 T | 7.55 | 0.75 |

**Supplemental Table 4: Brain FLAIR.** MRI intensity variance, skewness, and kurtosis measurements for the seven brain normalization methods across each intensity comparison. *Abbreviations: CSF = cerebral spinal fluid*.

| **Normalization Method** | **Comparison** | **Pooled St. Deviation** | **p-value** |
| --- | --- | --- | --- |
| **Intensity Variance** | | | |
| Unnormalized | Site 1 v Site 2 | 148539.11 | 1 |
|  | Site 1 v Site 3 | 159769.99 | 1 |
|  | Site 2 v Site 3 | 46779.06 | 0.88 |
| Standard Deviation | Site 1 v Site 2 | 0 | <0.001 |
|  | Site 1 v Site 3 | 0 | <0.001 |
|  | Site 2 v Site 3 | 0 | <0.001 |
| Z-Score | Site 1 v Site 2 | 0 | <0.001 |
|  | Site 1 v Site 3 | 0 | <0.001 |
|  | Site 2 v Site 3 | 0 | <0.001 |
| Min-Max | Site 1 v Site 2 | 0 | <0.001 |
|  | Site 1 v Site 3 | 0 | <0.001 |
|  | Site 2 v Site 3 | 0 | <0.001 |
| Scaled | Site 1 v Site 2 | 0 | <0.001 |
|  | Site 1 v Site 3 | 0 | <0.001 |
|  | Site 2 v Site 3 | 0 | <0.001 |
| CSF Mask | Site 1 v Site 2 | 0.12 | 0.01 |
|  | Site 1 v Site 3 | 0.12 | 0.35 |
|  | Site 2 v Site 3 | 0.07 | <0.001 |
| Tumor Mask | Site 1 v Site 2 | 0.02 | <0.001 |
|  | Site 1 v Site 3 | 0.02 | <0.001 |
|  | Site 2 v Site 3 | 0.01 | <0.001 |
| Unnormalized | GE v Siemens | 234842.29 | 0.6 |
| Standard Deviation | GE v Siemens | 0 | <0.001 |
| Z-Score | GE v Siemens | 0 | <0.001 |
| Min-Max | GE v Siemens | 0 | <0.001 |
| Scaled | GE v Siemens | 0.01 | <0.001 |
| CSF Mask | GE v Siemens | 0.08 | 0.97 |
| Tumor Mask | GE v Siemens | 0.02 | <0.001 |
| Unnormalized | 3 T v 1.5 T | 147472.22 | 1 |
| Standard Deviation | 3 T v 1.5 T | 0 | <0.001 |
| Z-Score | 3 T v 1.5 T | 0 | <0.001 |
| Min-Max | 3 T v 1.5 T | 0.01 | <0.001 |
| Scaled | 3 T v 1.5 T | 0.01 | <0.001 |
| CSF Mask | 3 T v 1.5 T | 0.11 | <0.001 |
| Tumor Mask | 3 T v 1.5 T | 0.02 | <0.001 |
| **Intensity Skewness** | | | |
| Unnormalized | Site 1 v Site 2 | 0.84 | 1 |
|  | Site 1 v Site 3 | 0.48 | 0.96 |
|  | Site 2 v Site 3 | 0.71 | 1 |
| Standard Deviation | Site 1 v Site 2 | 0.83 | 1 |
|  | Site 1 v Site 3 | 0.47 | 0.85 |
|  | Site 2 v Site 3 | 0.71 | 1 |
| Z-Score | Site 1 v Site 2 | 0.83 | 1 |
|  | Site 1 v Site 3 | 0.47 | 0.85 |
|  | Site 2 v Site 3 | 0.71 | 1 |
| Min-Max | Site 1 v Site 2 | 0.83 | 1 |
|  | Site 1 v Site 3 | 0.47 | 0.85 |
|  | Site 2 v Site 3 | 0.71 | 1 |
| Scaled | Site 1 v Site 2 | 0.83 | 1 |
|  | Site 1 v Site 3 | 0.47 | 0.85 |
|  | Site 2 v Site 3 | 0.71 | 1 |
| CSF Mask | Site 1 v Site 2 | 0.83 | 1 |
|  | Site 1 v Site 3 | 0.47 | 0.85 |
|  | Site 2 v Site 3 | 0.71 | 1 |
| Tumor Mask | Site 1 v Site 2 | 0.83 | 1 |
|  | Site 1 v Site 3 | 0.47 | 0.85 |
|  | Site 2 v Site 3 | 0.71 | 1 |
| Unnormalized | GE v Siemens | 0.47 | 0.29 |
| Standard Deviation | GE v Siemens | 0.47 | 0.29 |
| Z-Score | GE v Siemens | 0.47 | 0.29 |
| Min-Max | GE v Siemens | 0.47 | 0.29 |
| Scaled | GE v Siemens | 0.47 | 0.29 |
| CSF Mask | GE v Siemens | 0.47 | 0.29 |
| Tumor Mask | GE v Siemens | 0.47 | 0.29 |
| Unnormalized | 3 T v 1.5 T | 1.07 | 0.87 |
| Standard Deviation | 3 T v 1.5 T | 1.07 | 0.92 |
| Z-Score | 3 T v 1.5 T | 1.07 | 0.92 |
| Min-Max | 3 T v 1.5 T | 1.07 | 0.92 |
| Scaled | 3 T v 1.5 T | 1.07 | 0.92 |
| CSF Mask | 3 T v 1.5 T | 1.07 | 0.92 |
| Tumor Mask | 3 T v 1.5 T | 1.07 | 0.92 |
| **Intensity Kurtosis** | | | |
| Unnormalized | Site 1 v Site 2 | 4.12 | 1 |
|  | Site 1 v Site 3 | 1.21 | 1 |
|  | Site 2 v Site 3 | 3.31 | 1 |
| Standard Deviation | Site 1 v Site 2 | 4.12 | 1 |
|  | Site 1 v Site 3 | 1.21 | 1 |
|  | Site 2 v Site 3 | 3.31 | 1 |
| Z-Score | Site 1 v Site 2 | 4.12 | 1 |
|  | Site 1 v Site 3 | 1.21 | 1 |
|  | Site 2 v Site 3 | 3.31 | 1 |
| Min-Max | Site 1 v Site 2 | 4.12 | 1 |
|  | Site 1 v Site 3 | 1.21 | 1 |
|  | Site 2 v Site 3 | 3.31 | 1 |
| Scaled | Site 1 v Site 2 | 4.12 | 1 |
|  | Site 1 v Site 3 | 1.21 | 1 |
|  | Site 2 v Site 3 | 3.31 | 1 |
| CSF Mask | Site 1 v Site 2 | 4.12 | 1 |
|  | Site 1 v Site 3 | 1.21 | 1 |
|  | Site 2 v Site 3 | 3.31 | 1 |
| Tumor Mask | Site 1 v Site 2 | 4.12 | 1 |
|  | Site 1 v Site 3 | 1.21 | 1 |
|  | Site 2 v Site 3 | 3.31 | 1 |
| Unnormalized | GE v Siemens | 1.03 | 1 |
| Standard Deviation | GE v Siemens | 1.03 | 1 |
| Z-Score | GE v Siemens | 1.03 | 1 |
| Min-Max | GE v Siemens | 1.03 | 1 |
| Scaled | GE v Siemens | 1.03 | 1 |
| CSF Mask | GE v Siemens | 1.03 | 1 |
| Tumor Mask | GE v Siemens | 1.03 | 1 |
| Unnormalized | 3 T v 1.5 T | 3.81 | 0.82 |
| Standard Deviation | 3 T v 1.5 T | 3.82 | 0.85 |
| Z-Score | 3 T v 1.5 T | 3.82 | 0.85 |
| Min-Max | 3 T v 1.5 T | 3.82 | 0.85 |
| Scaled | 3 T v 1.5 T | 3.82 | 0.85 |
| CSF Mask | 3 T v 1.5 T | 3.82 | 0.85 |
| Tumor Mask | 3 T v 1.5 T | 3.82 | 0.85 |

**Supplemental Table 5: Brain ADC.** MRI intensity variance, skewness, and kurtosis measurements for the seven brain normalization methods across each intensity comparison. *Abbreviations: CSF = cerebral spinal fluid*.

| **Normalization Method** | **Comparison** | **Pooled St. Deviation** | **p-value** |
| --- | --- | --- | --- |
| **Intensity Variance** | | | |
| Unnormalized | Site 1 v Site 2 | 692150.21 | 1 |
|  | Site 1 v Site 3 | 820906.46 | 1 |
|  | Site 2 v Site 3 | 132467.01 | 1 |
| Standard Deviation | Site 1 v Site 2 | 0 | <0.001 |
|  | Site 1 v Site 3 | 0 | <0.001 |
|  | Site 2 v Site 3 | 0 | <0.001 |
| Z-Score | Site 1 v Site 2 | 0 | <0.001 |
|  | Site 1 v Site 3 | 0 | <0.001 |
|  | Site 2 v Site 3 | 0 | <0.001 |
| Min-Max | Site 1 v Site 2 | 0 | <0.001 |
|  | Site 1 v Site 3 | 0.01 | <0.001 |
|  | Site 2 v Site 3 | 0.01 | <0.001 |
| Scaled | Site 1 v Site 2 | 0.01 | <0.001 |
|  | Site 1 v Site 3 | 0.01 | <0.001 |
|  | Site 2 v Site 3 | 0.01 | <0.001 |
| CSF Mask | Site 1 v Site 2 | 0.11 | <0.001 |
|  | Site 1 v Site 3 | 0.16 | <0.001 |
|  | Site 2 v Site 3 | 0.07 | <0.001 |
| Tumor Mask | Site 1 v Site 2 | 0.22 | 0.02 |
|  | Site 1 v Site 3 | 0.27 | <0.001 |
|  | Site 2 v Site 3 | 0.11 | <0.001 |
| Unnormalized | GE v Siemens | 853108.65 | 0.67 |
| Standard Deviation | GE v Siemens | 0 | <0.001 |
| Z-Score | GE v Siemens | 0 | <0.001 |
| Min-Max | GE v Siemens | 0.01 | <0.001 |
| Scaled | GE v Siemens | 0.01 | <0.001 |
| CSF Mask | GE v Siemens | 0.17 | 0.37 |
| Tumor Mask | GE v Siemens | 0.28 | 0.24 |
| Unnormalized | 3 T v 1.5 T | 560811.48 | 0.81 |
| Standard Deviation | 3 T v 1.5 T | 0 | <0.001 |
| Z-Score | 3 T v 1.5 T | 0 | <0.001 |
| Min-Max | 3 T v 1.5 T | 0.01 | <0.001 |
| Scaled | 3 T v 1.5 T | 0.01 | <0.001 |
| CSF Mask | 3 T v 1.5 T | 0.13 | <0.001 |
| Tumor Mask | 3 T v 1.5 T | 0.2 | <0.001 |
| **Intensity Skewness** | | | |
| Unnormalized | Site 1 v Site 2 | 0.41 | <0.001 |
|  | Site 1 v Site 3 | 0.44 | 0.93 |
|  | Site 2 v Site 3 | 0.43 | 0.83 |
| Standard Deviation | Site 1 v Site 2 | 0.41 | <0.001 |
|  | Site 1 v Site 3 | 0.44 | 0.93 |
|  | Site 2 v Site 3 | 0.43 | 0.83 |
| Z-Score | Site 1 v Site 2 | 0.41 | <0.001 |
|  | Site 1 v Site 3 | 0.44 | 0.93 |
|  | Site 2 v Site 3 | 0.43 | 0.83 |
| Min-Max | Site 1 v Site 2 | 0.41 | <0.001 |
|  | Site 1 v Site 3 | 0.44 | 0.93 |
|  | Site 2 v Site 3 | 0.43 | 0.83 |
| Scaled | Site 1 v Site 2 | 0.41 | <0.001 |
|  | Site 1 v Site 3 | 0.44 | 0.93 |
|  | Site 2 v Site 3 | 0.43 | 0.83 |
| CSF Mask | Site 1 v Site 2 | 0.41 | <0.001 |
|  | Site 1 v Site 3 | 0.44 | 0.93 |
|  | Site 2 v Site 3 | 0.43 | 0.83 |
| Tumor Mask | Site 1 v Site 2 | 0.41 | <0.001 |
|  | Site 1 v Site 3 | 0.44 | 0.93 |
|  | Site 2 v Site 3 | 0.43 | 0.83 |
| Unnormalized | GE v Siemens | 0.46 | 0.61 |
| Standard Deviation | GE v Siemens | 0.46 | 0.61 |
| Z-Score | GE v Siemens | 0.46 | 0.61 |
| Min-Max | GE v Siemens | 0.46 | 0.61 |
| Scaled | GE v Siemens | 0.46 | 0.61 |
| CSF Mask | GE v Siemens | 0.46 | 0.61 |
| Tumor Mask | GE v Siemens | 0.46 | 0.61 |
| Unnormalized | 3 T v 1.5 T | 0.46 | 0.03 |
| Standard Deviation | 3 T v 1.5 T | 0.46 | 0.03 |
| Z-Score | 3 T v 1.5 T | 0.46 | 0.03 |
| Min-Max | 3 T v 1.5 T | 0.46 | 0.03 |
| Scaled | 3 T v 1.5 T | 0.46 | 0.03 |
| CSF Mask | 3 T v 1.5 T | 0.46 | 0.03 |
| Tumor Mask | 3 T v 1.5 T | 0.46 | 0.03 |
| **Intensity Kurtosis** | | | |
| Unnormalized | Site 1 v Site 2 | 1.87 | 0.82 |
|  | Site 1 v Site 3 | 2.52 | 0.67 |
|  | Site 2 v Site 3 | 2.2 | 0.08 |
| Standard Deviation | Site 1 v Site 2 | 1.87 | 0.82 |
|  | Site 1 v Site 3 | 2.52 | 0.67 |
|  | Site 2 v Site 3 | 2.2 | 0.08 |
| Z-Score | Site 1 v Site 2 | 1.87 | 0.82 |
|  | Site 1 v Site 3 | 2.52 | 0.67 |
|  | Site 2 v Site 3 | 2.2 | 0.08 |
| Min-Max | Site 1 v Site 2 | 1.87 | 0.82 |
|  | Site 1 v Site 3 | 2.52 | 0.67 |
|  | Site 2 v Site 3 | 2.2 | 0.08 |
| Scaled | Site 1 v Site 2 | 1.87 | 0.82 |
|  | Site 1 v Site 3 | 2.52 | 0.67 |
|  | Site 2 v Site 3 | 2.2 | 0.08 |
| CSF Mask | Site 1 v Site 2 | 1.87 | 0.82 |
|  | Site 1 v Site 3 | 2.52 | 0.67 |
|  | Site 2 v Site 3 | 2.2 | 0.08 |
| Tumor Mask | Site 1 v Site 2 | 1.87 | 0.82 |
|  | Site 1 v Site 3 | 2.52 | 0.67 |
|  | Site 2 v Site 3 | 2.2 | 0.08 |
| Unnormalized | GE v Siemens | 2.56 | 0.64 |
| Standard Deviation | GE v Siemens | 2.56 | 0.64 |
| Z-Score | GE v Siemens | 2.56 | 0.64 |
| Min-Max | GE v Siemens | 2.56 | 0.64 |
| Scaled | GE v Siemens | 2.56 | 0.64 |
| CSF Mask | GE v Siemens | 2.56 | 0.64 |
| Tumor Mask | GE v Siemens | 2.56 | 0.64 |
| Unnormalized | 3 T v 1.5 T | 2.18 | 0.5 |
| Standard Deviation | 3 T v 1.5 T | 2.18 | 0.5 |
| Z-Score | 3 T v 1.5 T | 2.18 | 0.5 |
| Min-Max | 3 T v 1.5 T | 2.18 | 0.5 |
| Scaled | 3 T v 1.5 T | 2.18 | 0.5 |
| CSF Mask | 3 T v 1.5 T | 2.18 | 0.5 |
| Tumor Mask | 3 T v 1.5 T | 2.18 | 0.5 |

**Supplemental Table 6: Breast MRI.** MRI intensity variance, skewness, and kurtosis measurements for the seven brain normalization methods across each intensity comparison.

| **Normalization Method** | **Comparison** | **Pooled St. Deviation** | **p-value** |
| --- | --- | --- | --- |
| **Intensity Variance** | | | |
| Unnormalized | Site 1 v Site 2 | 186386.46 | 0.75 |
|  | Site 1 v Site 3 | 195071.89 | 0.71 |
|  | Site 2 v Site 3 | 249715.17 | 0.83 |
| Standard Deviation | Site 1 v Site 2 | 0 | <0.001 |
|  | Site 1 v Site 3 | 0 | <0.001 |
|  | Site 2 v Site 3 | 0 | <0.001 |
| Z-Score | Site 1 v Site 2 | 0 | <0.001 |
|  | Site 1 v Site 3 | 0 | <0.001 |
|  | Site 2 v Site 3 | 0 | <0.001 |
| Min-Max | Site 1 v Site 2 | 0.01 | <0.001 |
|  | Site 1 v Site 3 | 0.01 | <0.001 |
|  | Site 2 v Site 3 | 0.01 | <0.001 |
| Scaled | Site 1 v Site 2 | 0.01 | <0.001 |
|  | Site 1 v Site 3 | 0.01 | <0.001 |
|  | Site 2 v Site 3 | 0.01 | <0.001 |
| Sternum Mask | Site 1 v Site 2 | 33.11 | 1 |
|  | Site 1 v Site 3 | 45 | 0.96 |
|  | Site 2 v Site 3 | 23.25 | 1 |
| Thorax Mask | Site 1 v Site 2 | 794.82 | 1 |
|  | Site 1 v Site 3 | 1066.45 | 1 |
|  | Site 2 v Site 3 | 538.78 | 1 |
| Unnormalized | GE v Siemens | 207220.1 | 1 |
| Standard Deviation | GE v Siemens | 0 | <0.001 |
| Z-Score | GE v Siemens | 0 | <0.001 |
| Min-Max | GE v Siemens | 0.01 | <0.001 |
| Scaled | GE v Siemens | 0.01 | <0.001 |
| Sternum Mask | GE v Siemens | 35.7 | 0.99 |
| Thorax Mask | GE v Siemens | 1005.07 | 1 |
| Unnormalized | 3 T v 1.5 T | 205673.7 | 1 |
| Standard Deviation | 3 T v 1.5 T | 0 | <0.001 |
| Z-Score | 3 T v 1.5 T | 0 | <0.001 |
| Min-Max | 3 T v 1.5 T | 0.01 | <0.001 |
| Scaled | 3 T v 1.5 T | 0.01 | <0.001 |
| Sternum Mask | 3 T v 1.5 T | 35.63 | 1 |
| Thorax Mask | 3 T v 1.5 T | 186386.46 | 0.75 |
| **Intensity Skewness** | | | |
| Unnormalized | Site 1 v Site 2 | 0.69 | 1 |
|  | Site 1 v Site 3 | 0.76 | 1 |
|  | Site 2 v Site 3 | 0.72 | 0.82 |
| Standard Deviation | Site 1 v Site 2 | 0.69 | 1 |
|  | Site 1 v Site 3 | 0.76 | 1 |
|  | Site 2 v Site 3 | 0.72 | 0.82 |
| Z-Score | Site 1 v Site 2 | 0.69 | 1 |
|  | Site 1 v Site 3 | 0.76 | 1 |
|  | Site 2 v Site 3 | 0.72 | 0.82 |
| Min-Max | Site 1 v Site 2 | 0.69 | 1 |
|  | Site 1 v Site 3 | 0.76 | 1 |
|  | Site 2 v Site 3 | 0.72 | 0.82 |
| Scaled | Site 1 v Site 2 | 0.69 | 1 |
|  | Site 1 v Site 3 | 0.76 | 1 |
|  | Site 2 v Site 3 | 0.72 | 0.82 |
| Sternum Mask | Site 1 v Site 2 | 0.69 | 1 |
|  | Site 1 v Site 3 | 0.76 | 1 |
|  | Site 2 v Site 3 | 0.72 | 0.82 |
| Thorax Mask | Site 1 v Site 2 | 0.69 | 1 |
|  | Site 1 v Site 3 | 0.76 | 1 |
|  | Site 2 v Site 3 | 0.72 | 0.82 |
| Unnormalized | GE v Siemens | 0.85 | 0.09 |
| Standard Deviation | GE v Siemens | 0.85 | 0.09 |
| Z-Score | GE v Siemens | 0.85 | 0.09 |
| Min-Max | GE v Siemens | 0.85 | 0.09 |
| Scaled | GE v Siemens | 0.85 | 0.09 |
| Sternum Mask | GE v Siemens | 0.85 | 0.09 |
| Thorax Mask | GE v Siemens | 0.85 | 0.09 |
| Unnormalized | 3 T v 1.5 T | 0.84 | 0.52 |
| Standard Deviation | 3 T v 1.5 T | 0.84 | 0.52 |
| Z-Score | 3 T v 1.5 T | 0.84 | 0.52 |
| Min-Max | 3 T v 1.5 T | 0.84 | 0.52 |
| Scaled | 3 T v 1.5 T | 0.84 | 0.52 |
| Sternum Mask | 3 T v 1.5 T | 0.84 | 0.52 |
| Thorax Mask | 3 T v 1.5 T | 0.84 | 0.52 |
| **Intensity Kurtosis** | | | |
| Unnormalized | Site 1 v Site 2 | 1.63 | 0.84 |
|  | Site 1 v Site 3 | 1.05 | 0.97 |
|  | Site 2 v Site 3 | 1.66 | 0.21 |
| Standard Deviation | Site 1 v Site 2 | 1.63 | 0.84 |
|  | Site 1 v Site 3 | 1.05 | 0.97 |
|  | Site 2 v Site 3 | 1.66 | 0.21 |
| Z-Score | Site 1 v Site 2 | 1.63 | 0.84 |
|  | Site 1 v Site 3 | 1.05 | 0.97 |
|  | Site 2 v Site 3 | 1.66 | 0.21 |
| Min-Max | Site 1 v Site 2 | 1.63 | 0.84 |
|  | Site 1 v Site 3 | 1.05 | 0.97 |
|  | Site 2 v Site 3 | 1.66 | 0.21 |
| Scaled | Site 1 v Site 2 | 1.63 | 0.84 |
|  | Site 1 v Site 3 | 1.05 | 0.97 |
|  | Site 2 v Site 3 | 1.66 | 0.21 |
| Sternum Mask | Site 1 v Site 2 | 1.63 | 0.84 |
|  | Site 1 v Site 3 | 1.05 | 0.97 |
|  | Site 2 v Site 3 | 1.66 | 0.21 |
| Thorax Mask | Site 1 v Site 2 | 1.63 | 0.84 |
|  | Site 1 v Site 3 | 1.05 | 0.97 |
|  | Site 2 v Site 3 | 1.66 | 0.21 |
| Unnormalized | GE v Siemens | 1.52 | 0.41 |
| Standard Deviation | GE v Siemens | 1.52 | 0.41 |
| Z-Score | GE v Siemens | 1.52 | 0.41 |
| Min-Max | GE v Siemens | 1.52 | 0.41 |
| Scaled | GE v Siemens | 1.52 | 0.41 |
| Sternum Mask | GE v Siemens | 1.52 | 0.41 |
| Thorax Mask | GE v Siemens | 1.52 | 0.41 |
| Unnormalized | 3 T v 1.5 T | 1.48 | 0.99 |
| Standard Deviation | 3 T v 1.5 T | 1.48 | 0.99 |
| Z-Score | 3 T v 1.5 T | 1.48 | 0.99 |
| Min-Max | 3 T v 1.5 T | 1.48 | 0.99 |
| Scaled | 3 T v 1.5 T | 1.48 | 0.99 |
| Sternum Mask | 3 T v 1.5 T | 1.48 | 0.99 |
| Thorax Mask | 3 T v 1.5 T | 1.48 | 0.99 |

**Supplemental Table 7: Radiomic feature average results.** Total count and average percentage of features that were statistically similar (i.e., p < 0.05) across the entire study. Note that ROI1 and ROI2 refer to the tissue-specific normalizations (i.e., prostate bladder/muscle, brain CSF/tumor, breast sternum/thorax).

| **Normalization** | **Radiomic Features** | **Stability (%)** | | | | | | | |
| --- | --- | --- | --- | --- | --- | --- | --- | --- | --- |
|  |  | **Prostate** | **T1** | **T1C** | **FLAIR** | **ADC** | **All Brain** | **Breast** | **All Tissue** |
| Unnormalized | Intensity | 26.8 | 21.2 | 22.4 | 18.4 | 20 | 20.5 | 18.4 | 21.7 |
|  | Texture | 44.1 | 36.8 | 37.8 | 40.6 | 39.7 | 38.7 | 41.6 | 40.5 |
|  | All | 39.5 | 32.6 | 33.7 | 34.7 | 34.5 | 33.9 | 35.5 | 35.5 |
| Standard Deviation | Intensity | 62.8 | 38.8 | 62.9 | 52.2 | 50.2 | 51 | 43.7 | 52.8 |
|  | Texture | 60.1 | 49.1 | 55.1 | 55.4 | 57.4 | 54.3 | 54.7 | 55.7 |
|  | All | 60.8 | 46.4 | 57.2 | 54.6 | 55.5 | 53.4 | 51.8 | 55 |
| Z-Score | Intensity | 82.4 | 47.3 | 54.3 | 49 | 51.8 | 50.6 | 51.8 | 58.5 |
|  | Texture | 75.5 | 55.3 | 55.7 | 50.6 | 54.6 | 54 | 59.7 | 60.1 |
|  | All | 77.3 | 53.2 | 55.4 | 50.2 | 53.8 | 53.1 | 57.6 | 59.7 |
| Min-Max | Intensity | 25.8 | 25.7 | 23.3 | 27.3 | 36.3 | 28.2 | 33.5 | 28.4 |
|  | Texture | 41.4 | 45.1 | 40.4 | 46 | 50.4 | 45.5 | 54.4 | 45.9 |
|  | All | 37.2 | 40 | 35.9 | 41.1 | 46.7 | 40.9 | 48.9 | 41.2 |
| Scaled | Intensity | 25.8 | 25.7 | 23.3 | 27.3 | 36.3 | 28.2 | 33.5 | 28.4 |
|  | Texture | 41.4 | 45.1 | 40.4 | 46 | 50.4 | 45.5 | 54.4 | 45.9 |
|  | All | 37.2 | 40 | 35.9 | 41.1 | 46.7 | 40.9 | 48.9 | 41.2 |
| ROI1 | Intensity | 50 | 43.7 | 65.3 | 36.7 | 38.8 | 46.1 | 22.9 | 43.5 |
|  | Texture | 57 | 45.3 | 60 | 45 | 42.1 | 48.1 | 47.4 | 50.1 |
|  | All | 55.1 | 44.9 | 61.4 | 42.8 | 41.2 | 47.6 | 40.9 | 48.4 |
| ROI2 | Intensity | 27.3 | 64.1 | 71 | 48.2 | 52.7 | 59 | 18.8 | 45.2 |
|  | Texture | 43.7 | 63.4 | 65.4 | 54 | 52.1 | 58.7 | 41.2 | 52.4 |
|  | All | 39.3 | 63.6 | 66.9 | 52.4 | 52.2 | 58.8 | 35.2 | 50.5 |
| All Images | Texture | 51.9 | 48.6 | 50.7 | 48.2 | 49.5 | 49.3 | 50.5 | 50.1 |
|  | GLCM | 63.4 | 60.6 | 63.5 | 60.6 | 63.4 | 62.1 | 62.4 | 62.4 |
|  | GLRLM | 46.6 | 42.1 | 44.7 | 42 | 40.2 | 42.3 | 42.7 | 43.4 |
|  | GLSZM | 39.7 | 38.9 | 38.9 | 36.1 | 39.5 | 38.3 | 40 | 38.9 |
|  | GLDZM | 40.5 | 40 | 39.1 | 36.6 | 36.3 | 38 | 38.9 | 38.7 |
|  | NGTDM | 52.9 | 54.9 | 54.3 | 50.9 | 53.1 | 53.3 | 57.7 | 53.9 |
|  | NGLDM | 49.8 | 40.7 | 45.2 | 45.2 | 47.1 | 44.5 | 48.7 | 46.4 |
|  | Intensity | 43 | 38.1 | 46.1 | 37 | 40.9 | 40.5 | 31.8 | 39.8 |
|  | Local Intensity | 17.9 | 1.4 | 22.9 | 14.3 | 14.3 | 13.2 | 7.1 | 13.4 |
|  | Intensity Based Statistics | 39.9 | 35.1 | 47.1 | 32.5 | 32.7 | 36.9 | 22.5 | 35.4 |
|  | Intensity Histogram | 46.9 | 41.5 | 46.8 | 39.9 | 48.4 | 44.2 | 36.4 | 43.6 |
|  | Intensity Volume Histogram | 45.5 | 46.2 | 47.6 | 47.1 | 45.2 | 46.5 | 50 | 46.8 |
|  | All Features | 49.5 | 45.8 | 49.5 | 45.3 | 47.2 | 46.9 | 45.5 | 47.4 |
